# Could graph neural networks learn better molecular representation for drug discovery? A comparison study of descriptor-based and graph-based models

**DOI:** 10.1186/s13321-020-00479-8

**Published:** 2021-02-17

**Authors:** Dejun Jiang, Zhenxing Wu, Chang-Yu Hsieh, Guangyong Chen, Ben Liao, Zhe Wang, Chao Shen, Dongsheng Cao, Jian Wu, Tingjun Hou

**Affiliations:** 1 Innovation Institute for Artificial Intelligence in Medicine of Zhejiang University, College of Pharmaceutical Sciences, Zhejiang University, Hangzhou, 310058 Zhejiang China; 2 State Key Lab of CAD & CG, Zhejiang University, Hangzhou, 310058 Zhejiang China; 3 College of Computer Science and Technology, Zhejiang University, Hangzhou, China; 4 Tencent Quantum Laboratory Tencent, Shenzhen, 518057 Guangdong China; 5 Shenzhen Institutes of Advanced Technology, Shenzhen, 518055 Guangdong China; 6 Xiangya School of Pharmaceutical Sciences, Central South University, Changsha, 410004 Hunan China

**Keywords:** Graph neural networks, Extreme gradient boosting, Ensemble learning, Deep learning, ADME/T prediction

## Abstract

Graph neural networks (GNN) has been considered as an attractive modelling method for molecular property prediction, and numerous studies have shown that GNN could yield more promising results than traditional descriptor-based methods. In this study, based on 11 public datasets covering various property endpoints, the predictive capacity and computational efficiency of the prediction models developed by eight machine learning (ML) algorithms, including four descriptor-based models (SVM, XGBoost, RF and DNN) and four graph-based models (GCN, GAT, MPNN and Attentive FP), were extensively tested and compared. The results demonstrate that on average the descriptor-based models outperform the graph-based models in terms of prediction accuracy and computational efficiency. SVM generally achieves the best predictions for the regression tasks. Both RF and XGBoost can achieve reliable predictions for the classification tasks, and some of the graph-based models, such as Attentive FP and GCN, can yield outstanding performance for a fraction of larger or multi-task datasets. In terms of computational cost, XGBoost and RF are the two most efficient algorithms and only need a few seconds to train a model even for a large dataset. The model interpretations by the SHAP method can effectively explore the established domain knowledge for the descriptor-based models. Finally, we explored use of these models for virtual screening (VS) towards HIV and demonstrated that different ML algorithms offer diverse VS profiles. All in all, we believe that the off-the-shelf descriptor-based models still can be directly employed to accurately predict various chemical endpoints with excellent computability and interpretability.
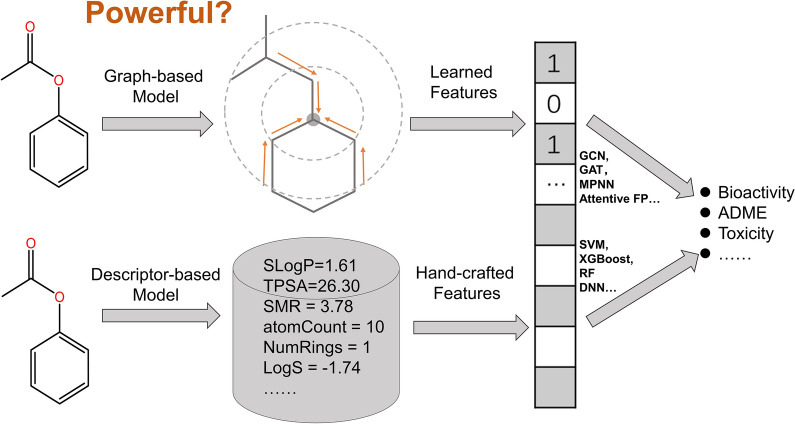

## Introduction

Molecular property modelling, which assists in hunting for chemicals with desired pharmacological and ADME/T (absorption, distribution, metabolism, excretion, and toxicity) properties, is one of the most classical cheminformatics tasks [1, 2]. A variety of machine learning (ML) approaches, such as Naive Bayes (NB) [3–5], k-Nearest Neighbors (k-NN) [6], logistic regression (LR) [7, 8], support vector machine (SVM) [9–13], random forest (RF), [10, 14, 15] artificial neural network (ANN) [13] and more, have been widely employed in property prediction. More recently, the emergence of deep learning (DL) methods has revolutionized this traditional cheminformatics task due to their extraordinary capacity to learn intricate relationships between structures and properties [16–23]. The models developed by DL can be roughly classified into two categories: descriptor-based models and graph-based models [24]. As to descriptor-based DL models, molecular descriptors and/or fingerprints commonly used in traditional quantitative structure–activity relationship (QSAR) models are used as the input, and then a specific DL architecture is employed to train a model [25]. As to graph-based DL models, the basic chemical information encoded by molecular graphs is used as the input, and then a graph-based DL algorithm, such as graph neural networks (GNN), is used to train a model. Similar to the convolutions on the regular data such as images and texts, GNN generalizes this operation to the irregular molecular graph that is a natural representation for chemical structures. More specifically, a graph *G* = (*V*, *E*) can be defined as the connectivity relations between a set of nodes (*V*) and a set of edges (*E*). Naturally, a molecule can also be considered as a graph consisting of a set of atoms (nodes) and a set of bonds (edges).

**Fig. 1:**
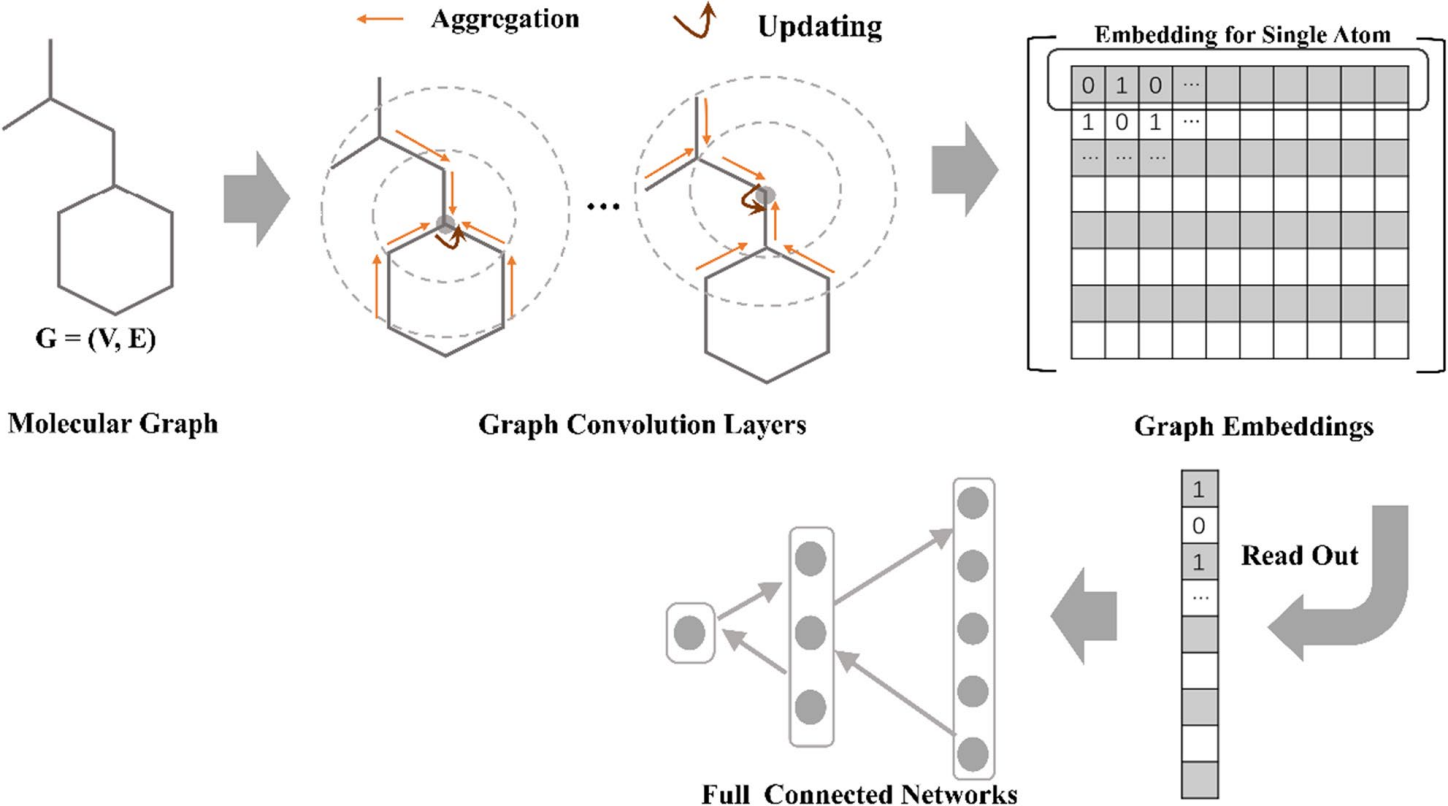
The general workflow of GNN in molecular property prediction

Essentially, GNN aims to learn the representations of each atom by aggregating the information from its neighboring atoms encoded by the atom feature vector and the information of the connected bonds encoded by the bond feature vector through message passing across the molecular graph recursively (Fig. 1), followed by the state updating of the central atoms and read-out operation. Then, the learned atom representations can be used for the prediction of molecular properties through the read-out phase [19, 26]. The key feature of GNN is its capacity to automatically learn task-specific representations using graph convolutions while does not need traditional hand-crafted descriptors and/or fingerprints. The state-of-the-art accuracy of GNN models in property prediction has been well represented [17, 24, 27–32]. The representative GNN models and their statistical performances on the MoleculeNet benchmark datasets [32] are summarized in Table 1. As we can see, their performances on the benchmark datasets vary from one to another, which may be attributed to the discrepancies on the model architectures, evaluation methods, training strategies and so on. Recently, a GNN method: Attentive FP, has gained increasing attention from the scientific community [27]. As shown in Table 1, Attentive FP yields the best predictions to 6 out of 11 benchmark datasets, including 2 regression tasks (ESOL and FreeSolv) and 4 classification tasks (MUV, BBBP, ToxCast and ClinTox), highlighting its impressive performance in modelling a variety of chemical properties in comparison with several other graph-based methods. A majority of those studies claimed that graph-based models are typically superior or comparable to traditional descriptor-based models [24, 30–35], and only a few studies gave the opposite conclusions [36]. For example, in 2017, Wu et al. reported MoleculeNet, a large benchmark for molecular machine learning, and the evaluation results illustrated that graph-based methods outperformed descriptor-based methods on most datasets [32]. Similarly, in 2019, Yang et al. introduced a novel GNN framework named directed message passing neural networks (D-MPNN), and the extensive evaluation on a large dataset collection indicated that D-MPNN consistently matched or outperformed descriptor-based methods on most datasets [24]. More recently, Korolev et al. reported a universal graph convolutional networks (GCN) architecture for the predictions of various chemical endpoints [33], and the application of GCN illustrated that its performance was comparable to state-of-the-art ML algorithms such as SVM, RF, and gradient boosting decision trees (GBDT).

**Table 1:** The reported GNN models in molecular property prediction

Year	Model Name	References	Datasets										
			Regression (RMSE)			Classification (AUC_ROC)							
			ESOL	FreeSolv	Lipop	MUV^a^	HIV	BACE	BBBP	Tox21	ToxCast	SIDER	ClinTox
2019	Attentive FP^b^	Xiong et al. [27]	*0.503 ± 0.076*	*0.736 ± 0.037*	0.578 ± 0.018	*0.221 ± 0.047*	0.832 ± 0.021	0.850 ± 0.012	*0.920 ± 0.015*	0.858 ± 0.014	*0.805 ± 0.022*	0.637 ± 0.017	*0.940 ± 0.018*
2019	D-MPNN^c^	Yang et al. [24]	0.665 ± 0.052	1.167 ± 0.150	0.596 ± 0.050	0.122 ± 0.020	0.816 ± 0.023	0.878 ± 0.032	0.913 ± 0.026	0.845 ± 0.015	0.737 ± 0.013	*0.646 ± 0.016*	0.894 ± 0.027
2019	PAGTN^d^	Chen et al. [29]	0.554 ± 0.060	NA	*0.572 ± 0.040*	NA	NA	*0.880 ± 0.010*	0.913 ± 0.030	NA	NA	NA	NA
2019	EIGNN^b^	Chen et al. [28]	0.653 ± 0.025	1.273 ± 0.137	0.776 ± 0.071	NA	NA	NA	NA	NA	NA	NA	NA
2018	EAGCN^b^	Shang et al. [30]	NA	0.950 ± 0.140	0.610 ± 0.020	NA	0.830 ± 0.010	NA	NA	*0.860 ± 0.010*	NA	NA	NA
2018	AGCN	Li et al [72].	NA	NA	NA	NA	NA	NA	NA	0.802	0.703	0.592	0.868
2017	GC^b^	Wu et al. [32]	0.970 ± 0.010	1.400 ± 0.160	0.655 ± 0.036	0.046 ± 0.031	0.763 ± 0.016^e^	0.783 ± 0.014^e^	0.690 ± 0.009^e^	0.829 ± 0.006	0.716 ± 0.014	0.638 ± 0.012	0.807 ± 0.047
2017	Weave^b^	Wu et al. [17, 32]	0.610 ± 0.070	1.220 ± 0.280	0.715 ± 0.035	0.109 ± 0.028	0.703 ± 0.039^e^	0.806 ± 0.002^e^	0.671 ± 0.014^e^	0.820 ± 0.010	0.742 ± 0.003	0.581 ± 0.027	0.832 ± 0.037
2017	DAG^b^	Wu et al. [32]	0.820 ± 0.080	1.630 ± 0.180	0.835 ± 0.039	NA	NA	NA	NA	NA	NA	NA	NA
2017	MPNN^b^	Wu et al. [32]	0.580 ± 0.030	1.150 ± 0.120	0.719 ± 0.031	NA	NA	NA	NA	NA	NA	NA	NA
2017	NA	Li et al [31]	NA	1.112	NA	NA	0.851	NA	NA	0.854	0.768	NA	NA

All the results were taken from the corresponding publication directly under the single model pattern and the best model for each dataset were italic

^a^Model built on MUV was evaluated by AUC-PRC (the area under precision-recall curve); ^b^Average performance of 3 times independent runs with the standard deviation; ^c^Average performance of 10 times independent runs with the standard deviation except for HIV, and HIV is the average performance of 3 times independent runs with the standard deviation; ^d^Average performance of 10 times independent runs with the standard deviation; ^e^Model was evaluated in scaffold splitting rather than random splitting; *NA* not available

In most of these reported studies, traditional ML models such as LR, RF, SVM (especially ‘gold standard’ RF) [31, 37] were employed to develop the prediction models based on a set of individual fingerprints (especially Extended Connectivity Fingerprints, ECFP) [31–33]. However, it is well known that the performance of descriptor-based models is highly depending on the descriptors used in training and many previous studies have highlighted that ML models only based on molecular fingerprints are not such well-performing [4, 5, 38, 39]. In addition, little attention was paid to several newly state-of-the-art ML algorithms, such as XGBoost and LightGBM, which have illustrated great potentials for modelling various molecular properties [39–42]. Accordingly, the conclusion that graph-based methods outperform traditional descriptor-based methods still remains controversial.

The present study attempts to give a comprehensive evaluation of descriptor-based and graph-based models on 11 public datasets with different property endpoints. Four ML algorithms were used to develop the descriptor-based models, including SVM, extreme gradient boosting (XGBoost), RF and deep neural networks (DNN). In order to better represent the chemical and structure features of the molecules for the descriptor-based models, the combination of one set of molecular descriptors (206 MOE 1-D and 2-D descriptors) and two sets of fingerprints (881 PubChem fingerprints and 307 substructure fingerprints) were considered, and such molecular representations are also commonly seen and easily accessible. Three typical GNN architectures (GCN, GAT and MPNN) and a state-of-the-art graph-based model (Attentive FP) were used as the graph-based model baselines, and the informationized molecular graph using atom-level or bond-level features were taken as the input. Both of the predictability and computability of these models were assessed. The results illustrate that the computational cost of the descriptor-based models is far less than that of the graph-based model baselines, and the descriptor-based models generally yield more promising predictions than the graph-based methods. More concretely, SVM generally performs best on the regression tasks. Both RF and XGBoost are reliable classifiers for the classification tasks, but the graph-based models, such as GCN and Attentive FP, can also show excellent performance on some tasks. In terms of computational cost, XGBoost and RF are efficient and they only need a few seconds to train a model even for a large dataset. Moreover, the established descriptor-based models were interpreted by the Shapley additive explanations (SHAP), and the important descriptors and structural features learned by the prediction models were highlighted. Finally, the developed ML models were used to conduct a virtual screening (VS) study toward human immunodeficiency virus (HIV), and the results indicate that different ML models offer varied performance in identifying potential HIV inhibitors. All in all, we believe that the ready-made and light-weight descriptor-based models can reach better or comparable accuracy, computability, and interpretability to the highly complicated and specialized graph-based DL models.

## Materials and methods

### Datasets

To well compare the performance of descriptor-based and graph-based models, the dataset collection related to drug discovery used by Attentive FP was also adopted in this study [27]. This dataset collection contains 11 different datasets originally reported in MoleculeNet for a variety of chemical endpoints [32]. In the study reported by Xiong et al. [27], the molecules that could not be successfully processed by RDKit [43] or the Attentive FP model were excluded from the original datasets. The details of those datasets are summarized in Table 2. Here, three datasets were used for the regression tasks, including ESOL, FreeSolv, and Lipop, and the remaining eight datasets were used for the classification tasks, which can be further divided into the single-task datasets (ESOL, FreeSolv, Lipop, HIV, BACE, and BBBP) and the multi-task datasets (CilnTox, SIDER, Tox21, ToxCast, and MUV). Notably, we found that, in the ToxCast multi-task datasets, some subdatasets are extremely imbalanced (the ratio of two classes is higher than 50) or quite small (the number of compounds is smaller than 500). Apparently, it seems reluctant to include these subdatasets for the development and assessment of ML models because of biased evaluation metric or insufficient training data, especially for traditional ML methods. One of the strengths for graph-based models is that multi-task learning can be applied for such highly imbalanced subdatasets and the corresponding statistics may be improved in comparison with traditional ML methods, but the prediction performances for such highly unbalanced subdatasets are not so convinced. Therefore, for the sake of fairness and simplification, such subdatasets were excluded directly, leading to the number of the tasks for ToxCast is 182, not the original number of 617. All the assessed ML models were evaluated based on the same remaining 182 ToxCast tasks, and we believe that the results can still make sense.

**Table 2:** The detailed information of the datasets used in this study

Datasets	Task Type	Compounds	Tasks	Metric	Descriptions
ESOL	Regression	1127	1	RMSE	Water solubility for organic small molecules
FreeSolv	Regression	639	1	RMSE	Hydration free energy of small molecules in water
Lipop	Regression	4200	1	RMSE	Octanol/water distribution coefficient (logD at pH = 7.4)
HIV	Classification	40748	1	AUC-ROC	Inhibition to HIV replication
BACE	Classification	1513	1	AUC-ROC	Inhibition to human β-secretase 1 (BACE-1)
BBBP	Classification	2035	1	AUC-ROC	Binary labels of blood–brain barrier penetration
ClinTox	Classification	1475	2	AUC-ROC	Qualitative data of drugs approved by the FDA and those that have failed clinical trials for toxicity reasons
SIDER	Classification	1366	27	AUC-ROC	Database of marketed drugs and adverse drug reactions (ADR), grouped into 27 system organ classes
Tox21	Classification	7811	12	AUC-ROC	Qualitative toxicity measurements on 12 biological targets, including nuclear receptors and stress response pathways
ToxCast	Classification	8539	182	AUC-ROC	Toxicology data for a large library of compounds based on in vitro high-throughput screening, including experiments on over 600 tasks
MUV	Classification	93087	17	AUC-PRC	Subset of PubChem BioAssay by applying a refined nearest neighbor analysis, designed for the validation of virtual screening techniques

### Molecular representation

Graph-based methods are capable of learning molecular representations by operating the convolutions on the encoded molecular graphs directly. In the graph representation for a molecule, the connectivity relation between atoms is represented by a graph *G *= (*V*, *E*). Here, the nodes *V* are represented by the node feature vector *X*_v_ consisting of a series of atomic features and the edges *E* are represented by the edge feature vector *E*_*km*_ consisting of a series of bond features, where the subscript *km* indicates that atoms *k* and *m* are bonded. Followed by previous studies [27], almost all the easily accessible atom/bond-level features were exhausted to comprehensively squeeze chemical information into molecular graph for graph-based models, where include nine kinds of atomic features (i.e., atom symbol, atom degree, formal charge, radical electrons, hybridization, aromaticity, hydrogens, chirality and chirality type) and four kinds of bond features (i.e., bond type, conjugation, ring, and stereo). Most of them were encoded into a molecular graph in a one-hot manner and subsequently the encoded molecular graph was used as the input. The more details about the molecular representations for graph-based models are available in the publication [27].

All the molecules were minimized using the MMFF94 force field in MOE (Version: 2015.1001) with the default parameters. Then, the expert-crafted descriptors and fingerprints were computed to develop the descriptor-based models. To comprehensively represent molecular structures, 206 MOE 1-D and 2-D descriptors and two sets of fingerprints, including 881 PubChem fingerprints (PubchemFP) and 307 substructure fingerprints (SubFP), were used. The MOE descriptors were calculated by MOE (Version: 2015.1001), and the two sets of fingerprints were calculated by PaDEL-Descriptor (Version: 2.1). [44] Prior to the development of the descriptor-based models, all the molecular features were pretreated as follows: (1) the features with missing values and extremely low variance (< 0.05) were removed; (2) the feature that has a high correlation (*r* > 0.95) with another feature was removed; (3) the retained features were normalized to the mean value of 0 and variance of 1.

### Machine learning algorithms

As one of the most classic cheminformatics problems, molecular property prediction has made considerable progress over the last decade due to the application of new ML methods represented by deep learning and ensemble learning [25, 40, 45, 46]. In this study, four representative ML algorithms (i.e., DNN, SVM, XGBoost and RF) were used to develop the descriptor-based models, and four representative graph-based methods (i.e., MPNN, GCN, GAT and Attentive FP) were employed to develop the graph-based models.

### Deep neural networks (DNN)

As one of the typical DL algorithms, DNN has an input layer, an output layer, and many hidden layers. DNN is composed of many individual neurons [16, 25]. Each neuron in DNN aggregates information from its connected neurons and then the aggregated information is activated by a non-linear activation function. Such manifestations mimick the behavior of biological neural networks. All the operations in DNN aim to learn intricate and rapidly-varying non-linear functions and extract a hierarchy of useful features from the input [18]. In this study, three hidden layers feed-forward neural networks were employed, and the following key hyper-parameters were optimized: L2 regularization (0 to 0.01), dropout rate (0.0 to 0.5) and neurons for each hidden layer (64, 128, 256, 512). The other important hyper-parameters were fixed: *ReLU* function that has been recommended by many previous studies was used as the activation function [25, 47], and the optimizer was set to an adaptive learning rate algorithm: *Adadelta* [48].

### Support vector machine (SVM)

SVM is one of the most popular ML approaches and it is appropriate for both classification and regression [9, 49, 50]. It is also capable of dealing with both linearly separable and linearly inseparable problems. For linearly inseparable feature space, the kernel trick is needed to map the original feature space onto a new higher separable linear space. The basic objective of SVM is to find the optimal hyperplane in the feature space that can maximize the distance between the data points and hyperplane, and the discriminant results generated from this optimal hyperplane should be insensitive to small perturbation of training samples. Here, the commonly used radial basis function (RBF) was used as the kernel and the following main hyper-parameters were optimized: C (0.1 to 100) and gamma values (0 to 0.2).

### Extreme gradient boosting (XGBoost)

XGBoost is one of the most representative ensemble learning ML algorithms under the frame of gradient boosting [51]. Compared with traditional gradient boosting, several algorithm optimizations were introduced to XGBoost, such as minor improvement in the loss function by penalizing the complexity of the model, introduction of shrinkage and column subsampling for further preventing over-fitting, employment of sparsity-aware split finding technique for efficient training on sparse data, etc. [51]. XGBoost has gained extensive attention in the property prediction due to its significant predictive power and low computational cost [42, 52, 53]. In the training of XGBoost, the following hyper-parameters were optimized: learning_rate (0.01 to 0.2), gamma (0 to 0.2), min_child_weight (1 to 6), subsample (0.7 to 1.0), colsample_bytree (0.7 to 1.0), max_depth (3 to 10) and n_estimators (50, 100, 200, 300, 400, 500, 1000).

### Random forest (RF)

Random forest is another representative ensemble learning ML algorithms. It constructs a strong classifier or regressor by an ensemble of individual decision trees under the frame of bagging and makes predictions by majority vote or averaging of multiple decision trees [10, 15]. In the implementation of RF algorithm, sample perturbation via bootstrap sampling of the training data and feature perturbation via random feature subset selection are introduced to improve the diversity of base learner (decision trees), which corrects for the overfitting habit of decision trees and subsequently enhances the generalization ability of RF. In the training of RF, the following hyper-parameters were optimized: n_estimators (10, 50, 100, 200, 300, 400, 500), max_depth (3 to 12), min_samples_leaf (1, 3, 5, 10, 20, 50), min_impurity_decrease (0 to 0.01) and max_features (‘sqrt’, ‘log2’, 0.7, 0.8, 0.9).

### Message passing neural networks (MPNN)

MPNN is a common framework for GNN that was used for chemical prediction in 2017 by Gilmer et al. [54], and it has shown versatility in many applications such as natural language processing, image segmentation, chemical/molecular graphs, and so on. Many recently proposed GNN architectures for molecular property prediction can be formulated in this flexible framework [24, 26, 34, 37]. In theory, MPNN operates the convolutions on undirected molecular graphs *G* = (*V*, *E*) with node features *X*_*v*_ and edge features *E*_*km*_. The forward propagation of MPNN has two phases: message passing phase and readout phase. The message passing phase transmits information across the molecular graph to learn a molecular embedding using the message functions *M*_*t*_ and node updating functions *U*_*t*_, and the readout phase computes a feature vector for the whole molecular graph using some readout functions *R* to model the properties of interest. More mathematical details are available in the study reported by Gilmer et al. [54] In the training of MPNN, the following hyper-parameters were optimized: L2 regularization (0, 10e-8, 10e-6, 10e-4), learning rate (10e-2.5, 10e-3.5, 10e-1.5), dimension of node feature in hidden layers (64, 32, 16), dimension of edge feature in hidden layers (64, 32, 16), and number of set2set layers (2,3,4). The number of message passing steps and set2set steps were fixed to 6.

### Graph convolutional networks (GCN)

To date, various GCN frameworks and variants have been proposed, and the most classical GCN model was introduced by Kipf et al. in 2017 [55]. Mathematically, it follows the propagation rule: $H^{{\left( {l + 1} \right)}} = \sigma \left( {\hat{D}^{{ - \frac{1}{2}}} \hat{A}\hat{D}^{{ - \frac{1}{2}}} H^{\left( l \right)} W^{\left( l \right)} } \right)$, where $H^{(l)}$ and $W^{(l)}$ denote the $l^{th}$ neural networks layer and its corresponding learnable parameters, respectively. $\sigma$ represents a non-linear activation function. Generally, *D* and *A* are the degree matrix and adjacency matrix, respectively, $\hat{A} = A + I$ where $I$ is the identity matrix, and $\hat{D}$ is the diagonal node degree matrix of $\hat{A}$. The design of the $\hat{D}^{{ - \frac{1}{2}}} \hat{A}\hat{D}^{{ - \frac{1}{2}}}$ term is intended to add a self-connection to each node and keep the scale of the feature vectors. From the message passing point of view, it can also be ascribed to the following two step: (1): aggregate neighbors’ information $h_{v}$ to produce an intermediate representation $\hat{h}_{u}$; (2) transform the aggregated representation $\hat{h}_{u}$ with a linear projection followed by a non-linearity activation: $h_{u} = \sigma \left( {W_{u} \hat{h}_{u} } \right)$. In this study, the vanilla GCN model proposed by Kipf et al. was used and the following hyper-parameters were optimized: L2 regularization (0, 10e−8, 10e−6, 10e−4), learning rate (10e−2.5, 10e−3.5, 10e−1.5), dimension of FNN classifier (64, 128, 256), and dimension of GCN hidden layers ([128, 128], [256, 256], [128, 64], [256, 128]).

### Graph attention network (GAT)

GAT is an extension of the vanilla GCN model, and the biggest distinction between vanilla GCN and GAT is the way of neighboring information aggregation. In the vanilla GCN model, the graph convolution operation aggregates the normalized sum of neighboring information. In the GAT, attention mechanisms by specifying different weights to different nodes are introduced and the corresponding graph convolution operation aggregates the weighed sum of neighboring information in a formulation: $H_{i}^{{\left( {l + 1} \right)}} = \sigma \left( {\sum_{j \in N\left( i \right)} \alpha_{ij}^{\left( l \right)} W^{\left( l \right)}H_{i}^{\left( l \right)} } \right)$, where $\alpha_{ij}^{\left( l \right)}$ is the normalized attention score between node $i$ and node $j$ in the $l^{th}$ graph convolution layer. $W, N\left( i \right)$ and $\sigma$ are learnable weight matrix, the neighbors of node $i$, and non-linear activation function respectively. The calculation of the attention score and other details can be reference to the corresponding publication [56]. The application of attention mechanisms in the graph convolution can force the model to learn the most meaningful parts in neighbors and local environment and it has gained preferable performance in comparison with other usual GCN architectures [27, 34, 56]. In the training of GAT model, the following key hyper-parameters were optimized for each task: L2 regularization (0, 10e−8, 10e−6, 10e−4), learning rate (10e−2.5, 10e−3.5, 10e−1.5), dimension of GAT hidden layers ([128, 128], [256, 256], [128, 64], [256, 128]), dimension of FNN classifier (64, 128, 256), and the number of attention heads ([2, 2], [3, 3], [4, 4], [3, 4], [2, 3]).

### Attentive FP

Attentive FP, proposed by Xiong et al. [27] is a state-of-the-art GNN model for molecular property prediction. In Attentive FP, the recursive neural networks (RNN) was employed to agglomerate the structural information encoding in molecular graph from nearby to distant and update the state of centered atom. Moreover, a graph attention mechanism was introduced to allow the model to focus on the most relevant parts of the input. The results reported by Xiong et al. illustrated that Attentive FP can achieve state-of-the-art predictions to a wide range of molecular properties (Table 1) [57]. The main hyper-parameters for Attentive FP include num_layers (the number of attentive layers for atom embedding), num_timesteps (the number of attentive layers for molecule embedding), graph_feat_size (fingerprint dimension), L2 regularization, learning rate, and dropout rate. Here, all those main hyper-parameters were optimized: L2 regularization (0, 10e-8, 10e-6, 10e-4), learning rate (10e-2.5, 10e-3.5, 10e-1.5), num_layers (2, 3, 4, 5, 6), num_timesteps (1, 2, 3, 4, 5), dropout (0, 0.1, 0.3, 0.5), and graph_feat_size (50, 100, 200, 300).

For the development of the four descriptor-based models, the DNN algorithm was implemented in the PyTorch package (Version: 1.3.1 + cu92) of Python (Version: 3.6.5 × 64), and the XGBoost (Version: 0.80), RF and SVM algorithms were implemented in the scikit-learn package (Version: 0.20.1) of Python [58]. All the four graph-based models were implemented by the Deep Graph Library (DGL) package (Version: 0.4.1) using PyTorch as the backend of Python [59].

### Model training, optimization and evaluation protocols

In the first stage, the same training, validation and test sets at a ratio of 8:1:1 used by Attentive FP were also used in our study (Additional file 1 generated from the source code provided in the github). For the assessed ML algorithms, the prediction on the validation set was used to guide the optimization of hyper-parameters. The Tree of Parzen Estimators (TPE) algorithm was used to identify the best hyper-parameters for different ML models in 50 evaluations (Here four graph-based models on the HIV and MUV datasets were in 30 evaluations due to the high computation overhead). The TPE algorithm is an optimization algorithm under the sequential model-based global optimization frame and capable of finding ideal hyper-parameters only through a few objective function evaluations. TPE was implemented by the hyperopt package (Version: 0.2) in Python (Version: 3.6.5 × 64) [60]. Then, in the second stage, in order to alleviate the effect of the randomness of data splitting, 50 independent runs with different random seeds for data splitting (training/validation/test = 8:1:1) were performed to evaluate each ML model in a more reliable way. Similarly, four graph-based models on the HIV and MUV datasets were in a 20 independent runs due to the high computation overhead, and the optimized hyper-parameters determined in the first stage were straightly adopted. For avoiding overfitting and tremendous time consumption, all the neural network (NN)-based model (i.e. DNN, GCN, GAT, MPNN and Attentive FP) were trained in an early stopping way for all tasks if no validation performance improvement was observed in successive 50 epochs, and followed by the previous DNN hyper-parameter recommendations [25, 61], the maximum epoch was set as an empirical value of 300 for all the task. The additional check of the training logs also proved that this empirical value is enough to learn representative parameters for NN-based models. The training batch for most tasks was set as 128. However, this number was also merely empirical and could change depending on the complexity of model and data volume. All the model training and evaluation scripts were available in Additional file 2.

According to the recommendations of MoleculeNet benchmarks [32], the classification models were evaluated by the area under the receiver operating characteristic curve (AUC-ROC) for the classification tasks except the maximum unbiased validation (MUV) dataset, which was evaluated by the area under precision-recall curve (AUC-PRC) due to its extreme biased data distribution. The regression models were evaluated by root mean square error (RMSE). In a more diverse evaluation, we also considered mean absolute error (MAE) and R-Square (R2) metrics for regression model. As shown in Table 2, five datasets contain more than one task. The multi-task learning was applied in the development of the five NN-based models including DNN, GCN, GAT, MPNN and Attentive FP for each multi-task dataset, and the average performance across multiple tasks was reported. However, it is not practical to generalize the multi-task learning to traditional descriptor-based models (i.e. SVM, XGBoost, and RF). In this case, each multi-task dataset was split into multiple single-task datasets and the individual descriptor-based model on each single dataset was trained, and then the average performance was reported in a similar way.

### Model interpretation

ML algorithms usually function as a “black-box”, and how to interpret these complicated ML models remains a big challenge. Several interpretation methods have been proposed to uncover the “black-box” essence of ML algorithms and they can be roughly classified into two major categories: model-specific and model-agnostic strategies. The model-specific strategies are relevant to the specific structure of a model, such as the feature weights for the simplistic linear model and feature importance determined by Gini index for RF model. One of the strengths for the model-agnostic strategies is that they do not depend on the specific model architecture and can mitigate the necessity to balance model performance and interpretability [62, 63]. Some model-agnostic strategies such as sensitivity analysis have been applied in model interpretation but it becomes inefficient with the increase of model dimensionality [64, 65].

Here, a recently-developed model-agnostic interpretation framework called SHapley Additive exPlanations (SHAP) was employed to interpret the ML models due to its both local and global interpretability [66]. SHAP method was inspired from the game theory and the corresponding SHAP value was employed to quantify the contributions of single players to a collaborative game [65]. Some published studies have demonstrated that SHAP method has high potential in understanding arbitrary complicated ML models [39, 65]. In a more specific way, this method defines an explanation model that belongs to a linear function of binary variables: $f\left( x \right) \approx g\left( {z^{\prime}} \right) = \emptyset_{0} + \mathop \sum \nolimits_{i = 1}^{M} \emptyset_{i} z_{i}^{'}$, where $z^{\prime} \in \left\{ {0, 1} \right\}^{M}$ denotes the absence (0) or presence (1) of a certain descriptor, and $M$ is the number of molecular descriptors. $\emptyset_{i}$ is the so-called SHAP value, and similar to previous descriptions, it measures the impact of the presence or absence of a descriptor on the model output, and the sum of all descriptor attributions $g\left( {z^{\prime}} \right)$ approximates the output $f\left( x \right)$ of the original model. More details about this method can be found in the relevant publications [39, 65]. The SHAP method was implemented in the shap package (Version: 0.35.0) of Python software (Version: 3.6.5 x64).

### Washing of the benchmark datasets

Data quality is one of the fundamental questions in cheminformatics and the incorrect or inappropriate structures contained in datasets would hinder the effort of developing reliable prediction models. Here we found that some salts, inorganics, counterions, solvents, mixtures and even duplicates with inconsistent labels existing in the datasets provided by Xiong et al. [27], but we do not remove them first for the sake of fairness. The original data was reported by Wu et al. and it is apparently unreasonable to use such datasets for model building. In this regard, we developed a python script based on MOE (Version: 2015.1001) and RDKit (Version: 2019.09.1) to automatically eliminate the incorrect or inappropriate structures from the original datasets with the following steps: (1) For the mixtures and compounds containing salts, counterions, and solvents, we used a compromised method of keeping the major component with the largest number of heavy atoms and the retained component was neutralized if possible. This step was accomplished by the *sdwash* module in MOE and the compounds that cannot be recognized by MOE were eliminated; (2) A molecule was identified as an inorganics if it does not contain any carbon atom and then eliminated from the datasets. Similarly, the compounds that cannot be recognized by RDKit were also eliminated in this step; (3) Duplicates were identified by the canonical SMILES generated from RDKit. After that, the duplicated records with inconsistent labels were removed.

## Results and discussion

### Performance of descriptor-based and graph-based models

At the outset, the same training, validation, and test sets for the development of the Attentive FP models were adopted, and the corresponding statistical results for the six single-task datasets including three regression tasks and three classification tasks given by the four descriptor-based and four graph-based models are summarized in Table 3 (regression tasks) and Table 4 (classification tasks).

**Table 3:** The performance comparison (RMSE) of the four descriptor-based and four graph-based models on the three regression datasets (data folds were generated from Attentive FP and the top three model were italic for each dataset)

Dataset	No.	Tasks	Metric	Model	Training	Validation	Test
ESOL	1127	1	RMSE	*SVM*	0.158	0.624	*0.516*
				XGBoost	0.188	0.511	0.571
				RF	0.391	0.635	0.631
				*DNN*	0.448	0.568	*0.553*
				GCN	0.429	0.622	0.598
				GAT	0.402	0.518	0.604
				MPNN	0.467	0.546	0.665
				*Attentive FP*	0.407	0.479	*0.471*
FreeSolv	639	1	RMSE	*SVM*	0.347	0.423	*0.674*
				*XGBoost*	0.106	0.685	*0.707*
				RF	0.536	0.932	0.888
				*DNN*	0.483	0.527	*0.724*
				GCN	0.187	0.526	0.795
				GAT	0.496	0.634	0.851
				MPNN	0.316	0.772	1.050
				Attentive FP	0.529	0.517	0.813
Lipop	4200	1	RMSE	*SVM*	0.185	0.552	*0.567*
				*XGBoost*	0.145	0.524	*0.556*
				RF	0.481	0.625	0.649
				DNN	0.210	0.553	0.591
				GCN	0.315	0.573	0.612
				GAT	0.409	0.602	0.676
				MPNN	0.474	0.606	0.662
				*Attentive FP*	0.282	0.521	*0.559*

**Table 4:** The performance comparison (AUC_ROC) of the four descriptor-based and four graph-based models on the three classification datasets (data folds were generated from Attentive FP and the top three model were italic for each dataset)

Dataset	No.	Tasks	Metric	Model	Training	Validation	Test
HIV	40748	1	AUC_ROC	SVM	1.000	0.821	0.840
				*XGBoost*	0.999	0.842	*0.848*
				RF	0.962	0.805	0.846
				*DNN*	0.978	0.835	*0.858*
				*GCN*	0.994	0.862	*0.857*
				GAT	0.997	0.853	0.825
				MPNN	0.968	0.865	0.828
				Attentive FP	0.905	0.852	0.847
BACE	1513	1	AUC_ROC	SVM	0.976	0.883	0.861
				*XGBoost*	1.000	0.898	*0.889*
				RF	0.989	0.876	0.861
				*DNN*	0.973	0.921	*0.883*
				GCN	1.000	0.945	0.876
				GAT	0.996	0.937	0.848
				MPNN	0.972	0.921	0.848
				*Attentive FP*	1.000	0.923	*0.889*
BBBP	2035	1	AUC_ROC	*SVM*	0.988	0.922	*0.899*
				XGBoost	0.977	0.946	0.886
				*RF*	0.991	0.929	*0.907*
				DNN	0.981	0.933	0.856
				GCN	0.997	0.947	0.881
				GAT	0.999	0.947	0.872
				MPNN	0.944	0.961	0.889
				*Attentive FP*	0.971	0.952	*0.907*

For the regression tasks, one of the graph-based models, Attentive FP, achieves the best statistical performance on ESOL with the RMSE of 0.471 for the test set, and the performances of SVM (RMSE = 0.516) and DNN (RMSE = 0.553) are slightly worse than it. As we can see, the performances of three classical graph-based models (i.e., GCN, GAT and MPNN) and RF are obviously unpleasant on this dataset. For FreeSolv, both SVM and XGBoost offer considerable and comparable performances with RMSE = 0.674 and 0.707 for the test set respectively, which are slightly better than that of DNN (RMSE = 0.724). With regard to Lipop, three methods including one graph-based method (Attentive FP) and two descriptor-based methods (SVM and XGBoost) achieve similar performances with RMSE ≈ 0.560 for the test set and this predictive capability is superior to other methods, especially RF, GAT and  MPNN. Clearly, the RF and three graph-based models (i.e., GCN, GAT and MPNN) show disappointing predictive capability to the three regression tasks. On average, SVM achieves the best predictions on the test sets of the regression tasks. XGBoost and Attentive FP perform similarly but slightly worse than SVM.

As for the three classification tasks including HIV, BACE and BBBP, it gets puzzled to tell which type of model, i.e. descriptor-based and graph-based, is superior in the light of statistical results only from one random partition. However, it can be observed that three descriptor-based models (i.e. XGBoost, RF, and DNN) and two graph-based models (i.e. GCN and Attentive FP) are more powerful than the other models in general. Concretely, GCN and DNN offer almost the same predictions to HIV with AUC-ROC ≈ 0.857 for the test set, and three models including XGBoost, Attentive FP and RF are slightly worse than them with AUC-ROC ≈ 0.847. Besides, XGBoost and Attentive FP give the same performances on BACE with AUC-ROC = 0.889 for the test set, and DNN is slightly inferior to them with AUC-ROC = 0.883 for the test set. For BBBP, both Attentive FP and RF offer the same predictive ability for the test set with AUC_ROC = 0.907, and SVM gives slightly worse results with AUC_ROC = 0.899 for the test set.

Next, the performances of the descriptor-based and graph-based models were further compared on the five multi-task datasets including ClinTox, SIDER, Tox21, ToxCast, and MUV. As shown in Table 5, it seems also struggling to distinguish which type of model is more promising. Here from the overall level, the models that perform well in the aforementioned three classification tasks (i.e. GCN, Attentive FP, XGBoost, RF and DNN) can still give satisfactory predictions to the five multi-task datasets. More specifically, for ClinTox, two descriptor-based models (SVM and RF) and one-graph based model (GAT) give more promising predictions than the other models. For both SIDER and Tox21, two descriptor-based models (XGBoost and RF) and one graph-based model (Attentive FP) share similar and more powerful predictions on the corresponding test sets. For MUV, one descriptor-based model (SVM) and two graph-based models (GAT and Attentive FP) offer more promising results on the test set compared with the others.

**Table 5:** The performance comparison (AUC_ROC, MUV: AUC_PRC) of the four descriptor-based and four graph-based models on the five multi-task classification datasets (data folds were generated from Attentive FP and the top three model were italic for each dataset)

Dataset	No.	Tasks	Metric	Model	Training	Validation	Test
ClinTox	1475	2	AUC_ROC	*SVM*	0.991	0.879	*0.966*
				XGBoost	0.997	0.954	0.919
				*RF*	0.972	0.939	*0.964*
				DNN	0.993	0.943	0.956
				GCN	0.987	0.967	0.901
				*GAT*	0.992	0.965	*0.968*
				MPNN	0.943	0.950	0.955
				Attentive FP	0.951	0.961	0.944
SIDER	1366	27	AUC_ROC	SVM	0.975	0.683	0.620
				*XGBoost*	0.930	0.732	*0.665*
				*RF*	0.934	0.678	*0.659*
				DNN	0.939	0.658	0.639
				GCN	0.940	0.697	0.647
				GAT	0.924	0.681	0.602
				MPNN	0.880	0.666	0.606
				*Attentive FP*	0.985	0.651	*0.670*
Tox21	7811	12	AUC_ROC	SVM	0.971	0.946	0.826
				*XGBoost*	0.990	0.885	*0.847*
				*RF*	0.981	0.861	*0.858*
				*DNN*	0.941	0.849	*0.854*
				GCN	0.992	0.857	0.837
				GAT	0.985	0.844	0.830
				MPNN	0.889	0.833	0.802
				Attentive FP	0.984	0.870	0.847
ToxCast	8539	182	AUC_ROC	SVM	0.987	0.731	0.724
				XGBoost	0.973	0.836	0.773
				RF	0.950	0.811	0.782
				*DNN*	0.950	0.910	*0.909*
				GCN	0.969	0.904	0.902
				GAT	0.975	0.905	0.904
				MPNN	0.860	0.858	0.849
				*Attentive FP*	0.990	0.921	*0.919*
MUV	93087	17	AUC_PRC	*SVM*	0.852	0.080	*0.144*
				XGBoost	0.730	0.158	0.087
				RF	0.707	0.061	0.091
				DNN	0.030	0.031	0.024
				GCN	0.115	0.063	0.052
				*GAT*	0.187	0.113	*0.134*
				MPNN	0.020	0.017	0.025
				*Attentive FP*	0.090	0.030	*0.141*

To our surprising, five NN-based models including DNN, GCN, GAT, MPNN and Attentive FP yields much better prediction than three descriptor-based models to the ToxCast dataset (average AUC-ROC = 0.897 for five NN-based models and 0.760 for three descriptor-based models). The careful analysis of the Attentive FP source code suggests that the unreasonable data splitting for ToxCast may attribute to the over-optimistic predictions of five NN-based models where multi-task learning was applied. More concretely, it is quite possible that all positive samples or negative samples may occur for some columns of the data folds generated from a strongly biased subdataset in ToxCast based on the random data splitting and the average AUC-ROC cannot be calculated for such data folds accordingly (AUC-ROC metric calculation error). In this case, Xiong et al. adopted a compromised splitting strategy where a stratified sampling at a ratio of 8:1:1 was individually applied to each single task of ToxCast to generate 182 independent training sets, validation sets and test sets for 182 different tasks [27]. After that, those independent training/validation/test sets were merged one task by one task in an outer join manner to produce the final training/validation/test set. It is the fact that the aforementioned situation (AUC-ROC metric calculation error) was well avoided, but the issue raised by such splitting strategy is the over-estimated statistical results when multi-task learning was applied because many samples in the final test or validation sets will be included in the final training set. However, such situation (over-estimated statistical results) was well evaded by descriptor-based model where each single task was detached to train the model individually and no duplicated samples could occur in the data folds. More details about the data splitting used by Xiong et al. could be found in their webpage [67]. Besides, it is the same manner for the splitting of the biased MUV dataset in Attentive FP. To our knowledge, a reasonable way to solve this problem is to change the random seed for data splitting if the randomly generated data folds suffer from such situation. Hence, the obvious inferiority of three descriptor-based models on ToxCast compared with five NN-based models may be reasonably explained by the over-optimistic predictions of our NN-based models (what we will discuss later).

Actually, it seems arbitrary to judge which of models is better only based on the statistical results from one-time run because of the randomness in data splitting. To evaluate the ML models in a more reliable way, 50 times independent runs based on different random seeds to split data into 50 different folds of training, validation, and test sets at the ratio of 8:1:1 were conducted for each dataset, and the average performance over the 50 folds with the corresponding standard deviation was used to evaluate the ML models. And the splitting strategy for the ToxCast and MUV datasets was revised. The corresponding statistical results for the 11 studied datasets given by eight assessed models are listed in Table 6 (three regression datasets), Table 7 (three single-task classification datasets) and Table 8 (five multi-task classification datasets). From the Table 8, it can be observed that the predictions to the randomly split ToxCast datasets (Table 5) are much worse than those to the data generated by the original splitting strategy used by Attentive FP (average AUC_ROC of five NN-based models: 0.897 to 0.770), demonstrating the over-optimistic predictions given by five NN-based models based on the original splitting strategy. Here, it can be found that the average performance of the 50 times independent runs is worse than that of the one-time run for the 11 studied datasets. To our knowledge, many previous studies evaluated the ML models by only averaging the performance from three independent runs and their results may be sensitive to the randomness of data splitting [27, 32]. To well illustrate this point, we counted the average performances for the top three runs and the worst three runs among the 50 times independent runs for XGBoost (Additional file 3: Table S1). It can be recognized that the average performances for the top three runs and the worst three runs have big discrepancies for XGBoost. Therefore, with the aim of alleviating the randomness of data splitting, it is recommended to conduct sufficient independent runs to evaluate ML models more reliably.

**Table 6:** The performance comparison (average RMSE) of the 50 times independent runs on the three regression datasets for the eight models. (the top three model were italic for each dataset)

Dataset	No.	Tasks	Metric	Model	Training	Validation	Test
ESOL	1127	1	RMSE	*SVM*	0.149 ± 0.005	0.565 ± 0.038	*0.569 ± 0.052*
				*XGBoost*	0.224 ± 0.057	0.573 ± 0.048	*0.582 ± 0.056*
				RF	0.391 ± 0.008	0.664 ± 0.053	0.663 ± 0.074
				DNN	0.492 ± 0.061	0.617 ± 0.060	0.670 ± 0.092
				GCN	0.272 ± 0.049	0.650 ± 0.064	0.708 ± 0.068
				GAT	0.300 ± 0.093	0.608 ± 0.083	0.658 ± 0.109
				MPNN	0.463 ± 0.074	0.652 ± 0.051	0.700 ± 0.073
				*Attentive FP*	0.390 ± 0.076	0.535 ± 0.045	*0.587 ± 0.065*
FreeSolv	639	1	RMSE	*SVM*	0.307 ± 0.023	0.804 ± 0.192	*0.852 ± 0.171*
				*XGBoost*	0.228 ± 0.168	0.988 ± 0.197	*1.025 ± 0.185*
				RF	0.518 ± 0.011	1.129 ± 0.248	1.143 ± 0.230
				*DNN*	0.574 ± 0.115	0.840 ± 0.158	*1.013 ± 0.197*
				GCN	0.703 ± 0.127	0.872 ± 0.191	1.149 ± 0.262
				GAT	0.937 ± 0.375	1.079 ± 0.204	1.304 ± 0.272
				MPNN	0.824 ± 0.220	1.130 ± 0.245	1.327 ± 0.279
				Attentive FP	0.720 ± 0.131	0.881 ± 0.207	1.091 ± 0.191
Lipop	4200	1	RMSE	*SVM*	0.191 ± 0.005	0.566 ± 0.037	*0.577 ± 0.039*
				*XGBoost*	0.191 ± 0.040	0.569 ± 0.033	*0.574 ± 0.034*
				RF	0.478 ± 0.003	0.660 ± 0.031	0.659 ± 0.031
				DNN	0.271 ± 0.068	0.583 ± 0.031	0.608 ± 0.034
				GCN	0.360 ± 0.081	0.616 ± 0.038	0.664 ± 0.086
				GAT	0.372 ± 0.084	0.658 ± 0.037	0.683 ± 0.060
				MPNN	0.476 ± 0.065	0.640 ± 0.037	0.673 ± 0.038
				*Attentive FP*	0.309 ± 0.045	0.533 ± 0.033	*0.553 ± 0.035*

**Table 7:** The performance comparison (Average AUC_ROC) of the 50 times independent runs on the three classification datasets for the eight models. (the top three model were italic for each dataset)

Dataset	No.	Tasks	Metric	Model	Training	Validation	Test
HIV	40748	1	AUC_ROC	*SVM*	1.000 ± 0.000	0.825 ± 0.023	*0.822 ± 0.020*
				XGBoost	0.990 ± 0.012	0.831 ± 0.022	0.816 ± 0.020
				RF	0.963 ± 0.002	0.819 ± 0.021	0.820 ± 0.016
				DNN	0.935 ± 0.040	0.825 ± 0.020	0.797 ± 0.018
				*GCN*	0.984 ± 0.024	0.852 ± 0.023	*0.834 ± 0.025*
				*GAT*	0.957 ± 0.036	0.841 ± 0.019	*0.826 ± 0.030*
				MPNN	0.934 ± 0.040	0.828 ± 0.022	0.811 ± 0.031
				Attentive FP	0.928 ± 0.052	0.839 ± 0.022	0.822 ± 0.026
BACE	1513	1	AUC_ROC	*SVM*	0.979 ± 0.002	0.891 ± 0.026	*0.893 ± 0.020*
				XGBoost	0.994 ± 0.010	0.903 ± 0.029	0.889 ± 0.021
				*RF*	0.988 ± 0.001	0.896 ± 0.031	*0.890 ± 0.022*
				DNN	0.976 ± 0.015	0.916 ± 0.024	0.890 ± 0.024
				*GCN*	0.990 ± 0.018	0.921 ± 0.025	*0.898 ± 0.019*
				GAT	0.981 ± 0.021	0.916 ± 0.024	0.886 ± 0.023
				MPNN	0.926 ± 0.028	0.876 ± 0.030	0.838 ± 0.027
				Attentive FP	0.970 ± 0.029	0.906 ± 0.033	0.876 ± 0.023
BBBP	2035	1	AUC_ROC	SVM	0.988 ± 0.002	0.919 ± 0.029	0.919 ± 0.028
				*XGBoost*	0.995 ± 0.005	0.938 ± 0.022	*0.926 ± 0.026*
				*RF*	0.990 ± 0.001	0.929 ± 0.026	*0.927 ± 0.025*
				*DNN*	0.990 ± 0.010	0.938 ± 0.022	*0.922 ± 0.029*
				GCN	0.981 ± 0.018	0.931 ± 0.024	0.903 ± 0.027
				GAT	0.987 ± 0.016	0.927 ± 0.022	0.898 ± 0.033
				MPNN	0.961 ± 0.024	0.916 ± 0.030	0.879 ± 0.037
				Attentive FP	0.972 ± 0.021	0.922 ± 0.027	0.887 ± 0.032

**Table 8:** The performance comparison (Average AUC_ROC, MUV: Average AUC_PRC) of the 50 times independent runs on the five multi-task classification datasets for the eight models. (the top three model were italic for each dataset)

Dataset	No.	Tasks	Metric	Model	Training	Validation	Test
ClinTox	1475	2	AUC_ROC	SVM	0.922 ± 0.001	0.896 ± 0.048	0.888 ± 0.044
				*XGBoost*	0.985 ± 0.009	0.938 ± 0.035	*0.911 ± 0.036*
				*RF*	0.975 ± 0.003	0.918 ± 0.041	*0.911 ± 0.042*
				DNN	0.984 ± 0.014	0.929 ± 0.041	0.884 ± 0.051
				GCN	0.977 ± 0.020	0.945 ± 0.039	0.895 ± 0.046
				GAT	0.989 ± 0.010	0.941 ± 0.033	0.888 ± 0.042
				MPNN	0.895 ± 0.056	0.884 ± 0.069	0.847 ± 0.062
				*Attentive FP*	0.965 ± 0.018	0.943 ± 0.033	*0.904 ± 0.043*
SIDER	1366	27	AUC_ROC	SVM	0.953 ± 0.021	0.630 ± 0.025	0.630 ± 0.021
				*XGBoost*	0.954 ± 0.010	0.694 ± 0.023	*0.642 ± 0.020*
				*RF*	0.932 ± 0.001	0.655 ± 0.024	*0.646 ± 0.022*
				DNN	0.814 ± 0.064	0.657 ± 0.029	0.631 ± 0.028
				*GCN*	0.902 ± 0.047	0.656 ± 0.021	*0.634 ± 0.026*
				GAT	0.865 ± 0.068	0.663 ± 0.024	0.627 ± 0.024
				MPNN	0.741 ± 0.010	0.637 ± 0.030	0.598 ± 0.031
				Attentive FP	0.834 ± 0.103	0.657 ± 0.024	0.623 ± 0.026
Tox21	7811	12	AUC_ROC	SVM	0.972 ± 0.001	0.821 ± 0.013	0.817 ± 0.009
				XGBoost	0.989 ± 0.005	0.857 ± 0.009	0.836 ± 0.010
				*RF*	0.981 ± 0.001	0.840 ± 0.010	*0.838 ± 0.011*
				*DNN*	0.920 ± 0.022	0.849 ± 0.012	*0.840 ± 0.014*
				GCN	0.961 ± 0.019	0.846 ± 0.013	0.836 ± 0.016
				GAT	0.946 ± 0.025	0.842 ± 0.013	0.835 ± 0.014
				MPNN	0.896 ± 0.023	0.826 ± 0.014	0.809 ± 0.017
				*Attentive FP*	0.939 ± 0.021	0.859 ± 0.012	*0.852 ± 0.012*
ToxCast	8539	182	AUC_ROC	SVM	0.982 ± 0.007	0.723 ± 0.005	0.722 ± 0.006
				XGBoost	0.976 ± 0.002	0.800 ± 0.004	0.774 ± 0.004
				*RF*	0.949 ± 0.000	0.783 ± 0.005	*0.782 ± 0.005*
				*DNN*	0.900 ± 0.021	0.797 ± 0.017	*0.786 ± 0.019*
				GCN	0.891 ± 0.020	0.784 ± 0.019	0.770 ± 0.016
				GAT	0.881 ± 0.021	0.782 ± 0.018	0.768 ± 0.018
				MPNN	0.802 ± 0.033	0.746 ± 0.022	0.731 ± 0.021
				*Attentive FP*	0.921 ± 0.037	0.804 ± 0.020	*0.794 ± 0.017*
MUV	93087	17	AUC_PRC	*SVM*	0.834 ± 0.046	0.107 ± 0.036	*0.112 ± 0.045*
				*XGBoost*	0.646 ± 0.064	0.095 ± 0.039	*0.068 ± 0.028*
				RF	0.704 ± 0.019	0.053 ± 0.024	0.061 ± 0.032
				DNN	0.027 ± 0.028	0.030 ± 0.031	0.021 ± 0.030
				GCN	0.182 ± 0.012	0.067 ± 0.030	0.061 ± 0.034
				GAT	0.151 ± 0.078	0.062 ± 0.028	0.057 ± 0.030
				MPNN	0.011 ± 0.005	0.024 ± 0.022	0.016 ± 0.010
				Attentive FP	0.066 ± 0.052	0.040 ± 0.034	0.038 ± 0.024

As shown in the Table 6, it can be recognized that two descriptor-based models (SVM and XGBoost) and one graph-based model (Attentive FP) generally give better performances than the other models, which is consistent, to some extent, with the findings from the previous one random split. Among them, SVM gives the best predictions to the ESOL and FreeSolv datasets with average RMSE of 0.569 and 0.852 to the test sets, respectively. Attentive FP gives the best predictions to the Lipop dataset with average RMSE of 0.553 to the test set, and SVM and XGBoost are slightly worse than Attentive FP with RMSE ≈ 0.574. Here, XGBoost offers satisfactory but slightly worse predictions to all the three regression datasets (average RMSE = 0.582, 1.025, and 0.574 to ESOL, Freesolv, and Lipop respectively) compared with SVM and Attentive FP. In addition, the MAE and R2 metrics given by the eight models on the three regression tasks were also calculated (Additional file 3: Tables S2 and S3). As shown in Additional file 3: Table S2 and S3, similar conclusions could be drawn where SVM, XGBoost and Attentive FP are well-performing regressors and on average SVM is the best one. Here what we found from Table 6 is that the descriptor-based models, especially SVM, generally show much better training set performances in comparison with the graph-based models (especially for two smallest datasets FreeSolv and ESOL). However, some graph-based models, especially Attentive FP, are able to reach comparable prediction results to the descriptor-based models for the test sets, implying that the descriptor-based models are more likely to be over-fitted and less generalized compared with the graph-based models learnt from small and chemically narrow datasets. As for the three single-task classification datasets shown in Table 7, what we can find is that the four descriptor-based models are obviously superior to the four graph-based models on the BBBP dataset, where the average AUC_ROC of the four descriptor-based models is 0.924 compared with that of 0.891 for the four graph-based models. Similarly, on average the four descriptor-based models can give more reliable predictions to the BACE dataset where the average AUC_ROC of the four descriptor-based models is 0.891 compared with that of 0.875 for the four graph-based models. However, for the larger HIV, it seems that the graph-based models are slightly better than the descriptor-based models, implying that inclusion of more samples may be helpful to train a better graph-based model. In some cases, one may need to re-train their ML models with the gradual accumulation of available experimental datasets. Such operations can benefit more to graph-based models due to their data-hungry essence, but the rapid accumulation of qualitied experimental datasets is not an easy task. On the contrary, regular re-training of ML models by adding a small number of new compounds one time could be some of routine. Generally speaking, the optimization of hyper-parameters is necessary when re-training ML models, especially for NN-based models where their performances are sensitive to the hyper-parameters such as the initial parameters and learning rate. Compared with graph-based models, descriptor-based models such as RF or SVM may be more stable for a long time. With regard to the five multi-task datasets shown in Table 8, it can be found that the descriptor-based models, especially XGBoost and RF, achieve better predictions than the graph-based models on the ClinTox, SIDER and MUV datasets. However, one graph-based model, Attentive FP, achieves the best predictions to the two relatively large toxicity-relevant datasets including Tox21 and ToxCast with average AUC_ROC of 0.852 and 0.794 to the test sets, respectively, which may benefit from the multi-task learning and larger data volume. Numerous studies demonstrated that multi-task models have advantages over single-task models due to their ability to excavate the inconspicuous hidden relations between different subtasks and transparently share the learned features among all the tasks [57, 68, 69]. Nevertheless, the performance of multi-task models is highly related to the favorable correlations of individual tasks but such ready-to-use tasks are not so commonly seen in practical drug discovery campaigns. For the purpose of simplicity, we counted the top three models and the corresponding performances based on the results from 50 times independent runs for each dataset. As can be seen from Table 9, the descriptor-based model achieves the best predictions to six out of 11 datasets including ESOL, FreeSolv, BBBP, ClinTox, SIDER and MUV. Moreover, it can be observed that the top three models of all the datasets were mainly occupied by the descriptor-based models (the ratio is 24/33 = 73%), substantiating the more powerful predictive abilities of the descriptor-based models compared with the graph-based models. It is possible that the superiority of the descriptor-based models for some datasets (ESOL, FreeSolv, and Lipop) may be partially contributed from the descriptors that are highly correlated to the target values (such as the ‘LogS’ descriptor for the ESOL dataset). To systematically check this problem, we removed the top three descriptors that are highly correlated to the target values according to the Pearson’s correlation coefficients (ESOL: ‘logS’, ‘h_logS’, and ‘SlogP’; FreeSolv: ‘vsa_pol’, ‘h_emd’ and ‘a_donacc’; Lipop: ‘SlogP’, ‘h_logD’, and ‘logS’) and then used the remaining descriptors to reconstruct the four descriptor-based models based on the optimal hyper-parameter configurations determined in the first evaluation stage. The evaluation metrics were also averaged from the 50 times independent runs (Additional file 3: Table S4). It can be observed that the performance of the models developed based on the remaining descriptors do not show large difference compared with those developed based on the original descriptors. Moreover, we found that the descriptor-based models without thse high-related descriptors are still superior to the graph-based models (Additional file 3: Table S4). Here what we found is that the graph-based models can outperform the descriptor-based models on some lager or multi-task datasets such as the HIV, Tox21 and ToxCast datasets, which is in well accordance with the previous conclusions where DNN excel at larger amounts of data and multi-task learning [68, 69]. However, to build such generalizable and robust deep models requires large-scale high-quality datasets and the datasets in the practical drug discovery campaigns routinely suffer from narrow chemical diversity and insignificant sample sizes [70]. On the ground, we believe that the descriptor-based models can be still widely used and give reliable predictions in the drug discovery campaigns.

**Table 9:** The top three model and corresponding performances based on the results from 50 times independent runs for each dataset. (the descriptor-based models were colored as italic and the graph-based model were colored as undeline)

Dataset	No.	Tasks	Metric	Top 1	Top 2	Top 3
ESOL	1127	1	RMSE	*SVM (0.569 ± 0.052)*	*XGBoost (0.582 ± 0.056)*	Attentive FP (0.587 ± 0.065)
FreeSolv	639	1	RMSE	*SVM (0.852 ± 0.171)*	*DNN (1.013 ± 0.197)*	*XGBoost (1.025 ± 0.185)*
Lipop	4200	1	RMSE	Attentive FP (0.553 ± 0.035)	*XGBoost (0.574 ± 0.034)*	*SVM (0.577 ± 0.039)*
HIV	40748	1	AUC_ROC	GCN (0.834 ± 0.025)	GAT (0.826 ± 0.030)	*SVM (0.822 ± 0.020)*
BACE	1513	1	AUC_ROC	GCN (0.898 ± 0.019)	*SVM (0.893 ± 0.020)*	*RF (0.890 ± 0.022)*
BBBP	2035	1	AUC_ROC	*RF (0.927 ± 0.025)*	*XGBoost (0.926 ± 0.026)*	*DNN (0.922 ± 0.029)*
ClinTox	1475	2	AUC_ROC	*XGBoost (0.911 ± 0.036)*	*RF (0.911 ± 0.042)*	Attentive FP (0.904 ± 0.043)
SIDER	1366	27	AUC_ROC	*RF (0.646 ± 0.022)*	*XGBoost (0.642 ± 0.020)*	GCN (0.634 ± 0.026)
Tox21	7811	12	AUC_ROC	Attentive FP (0.852 ± 0.012)	*DNN (0.840 ± 0.014)*	*RF (0.838 ± 0.011)*
ToxCast	8539	182	AUC_ROC	Attentive FP (0.794 ± 0.017)	*DNN (0.786 ± 0.019)*	*RF (0.782 ± 0.005)*
MUV	93087	17	AUC_PRC	*SVM (0.112 ± 0.045)*	*XGBoost (0.068 ± 0.028)*	*RF (0.061 ± 0.032)*

In conclusion, regardless of the statistical results on the same data folds used by Attentive FP or a more reliable 50 times independent runs, what we found is that the traditional descriptor-based models generally outperform the state-of-the-art graph-based models. Among them, SVM is the best algorithm in modelling regression tasks. Both RF and XGBoost can be well-performing in modelling classification tasks, and some graph-based models, such as Attentive FP and GCN, can outperform the descriptor-based model on some larger or multi-task datasets.

### Computational consumption of different ML algorithms

It is worthwhile mentioning that an optimal predictive model should have a good balance between prediction accuracy and computational efficiency. As we all know, the run time complexity of SVM is quadratic to the number of training data [36]. As can be seen from Table 10, it takes a few seconds (average wall-clock time) to fit a model for the tasks whose data size is less than 4000. However, the average wall-clock time is centupled when fitting the largest HIV dataset (data size of 40,748). That is to say, SVM is a good choice in dealing with small to medium datasets, but it will be frustrated when dealing with large datasets. To some extent, the same problem exists for the NN-based methods, which highly depend on the acceleration of graphics processing units (GPU) cards. However, XGBoost and RF provide a parallel tree training with high efficiency, and one of their strengths is the speed [40].

**Table 10:** The mean wall-clock time (seconds) for the six single-task datasets given by the four descriptor-based and four graph-based models

Dataset	SVM^a^	XGBoost^b^	RF^b^	DNN^c^	GCN^d^	GAT^d^	MPNN^d^	Attentive FP^d^
FreeSolv (639)	0.17	0.209	1.429	6.27	18.458	29.37	77.85	20.927
ESOL (1127)	0.51	0.329	0.342	9.032	68.197	80.597	181.114	59.199
Lipop (4200)	6.431	7.379	5.722	28.686	159.879	151.191	611.048	652.777
BACE (1513)	2.105	0.327	1.327	8.911	108.967	156.074	630.748	137.291
BBBP (2035)	8.033	0.242	0.873	6.74	83.062	129.817	316.224	98.743
HIV (40748)	852.312	23.653	14.118	215.965	867.148	1122.126	1867.602	677.536

^a^SVM was implemented with the scikit-lear*n* package and run in a single thread (CPU: Intel(R) Xeon(R) CPU E5-2620 v2 @ 2.10 GHz); ^b^XGBoost and RF were implemented with the scikit-learn package and run in six parallel threads (CPU: Intel(R) Xeon(R) CPU E5-2620 v2 @ 2.10 GHz); ^c^DNN was implemented with PyTorch package and run in a single GPU card (NVIDIA GEFORCE RTX 2080 Ti with video memory of 11G); ^d^GCN, GAT, MPNN and Attentive FP were implemented with DGL package using PyTorch as the backend and run in a single GPU card (NVIDIA GEFORCE RTX 2080 Ti with video memory of 11G); All tested NN-based models were trained with a batch-size 128 in early-stopping way as described in ‘Materials and methods’ (HIV with a batch-size 128*5 due to the large data volume)

Here, we summarized the training speed of the four descriptor-based and four graph-based models on the six single-task datasets (Table 10), and the training speed was evaluated by the mean wall-clock time (seconds) from five independent runs where each run is to fit one corresponding model using the corresponding optimal hyper-parameters. It is worthwhile that the training speed of ML models can partly depend on the used hyper-parameters, such as the hidden layers of DNN, the trees of RF model and the graph convolution layers of GNN model. In this study, the training speed of all the ML models were evaluated under the corresponding optimal hyper-parameters determined in the first stage of performance comparison. In addition, we shall emphasize that we are not analyzing the time and space complexity of different algorithms theoretically but intend to provide intuitive and touchable elapsed time of different algorithms under the affordable computational resources. All the compared algorithms were implemented by the recognized python packages (i.e., scikit-learn, PyTorch and PyTorch-based DGL), and more details can be accessed from the footnote of Table 10. The choice of one-core, multi-cores or GPU largely depends on the inherent nature and common usage scenarios of algorithms, and what we try to present here is more likely a kind of rough users’ experience under the common usage scenarios, not the exactly CPU or GPU-time.

As shown in Table 10, the training speed for the descriptor-based models is overwhelmingly faster than that of the graph-based models. For the three traditional descriptor-based models, only a few seconds were needed to finish the training of a model to most datasets. Among them, XGBoost and RF are the two most efficient algorithm and they are also able to manage big data with high proficiency. As expected, SVM performs efficiently on the relatively small datasets but its practicability will become much worse for large datasets. The descriptor-based DNN models show higher computability than GCN, GAT, MPNN and Attentive FP, but all the NN-based models are highly dependent on GPU acceleration as mentioned above. Here, the top-performing graph-based algorithm, Attentive FP, demonstrates affordable computational efficiency compared with its counterparts. Among the four graph-based models, the vanilla GCN model is the most efficient algorithm and MPNN model is the worst one, which is in line with the common sense where the frameworks of vanilla GCN model are much simpler than that of MPNN model. Actually, the total wall-clock time including the hyper-parameter selection for each model was also analyzed but the conclusions are basically similar to the results discussed above (data are not shown).

Briefly, in terms of computational cost, the descriptor-based models are basically more efficient than the graph-based models. Among them, XGBoost and RF give the best computational efficiency and it only needs a few seconds to train a model even for a large dataset. The descriptor-based DNN method is the most efficient one in its counterparts including GCN, GAT, MPNN and Attentive FP, but the training of them largely depends on GPU acceleration.

### The interpretation of XGBoost Model

**Fig. 2:**
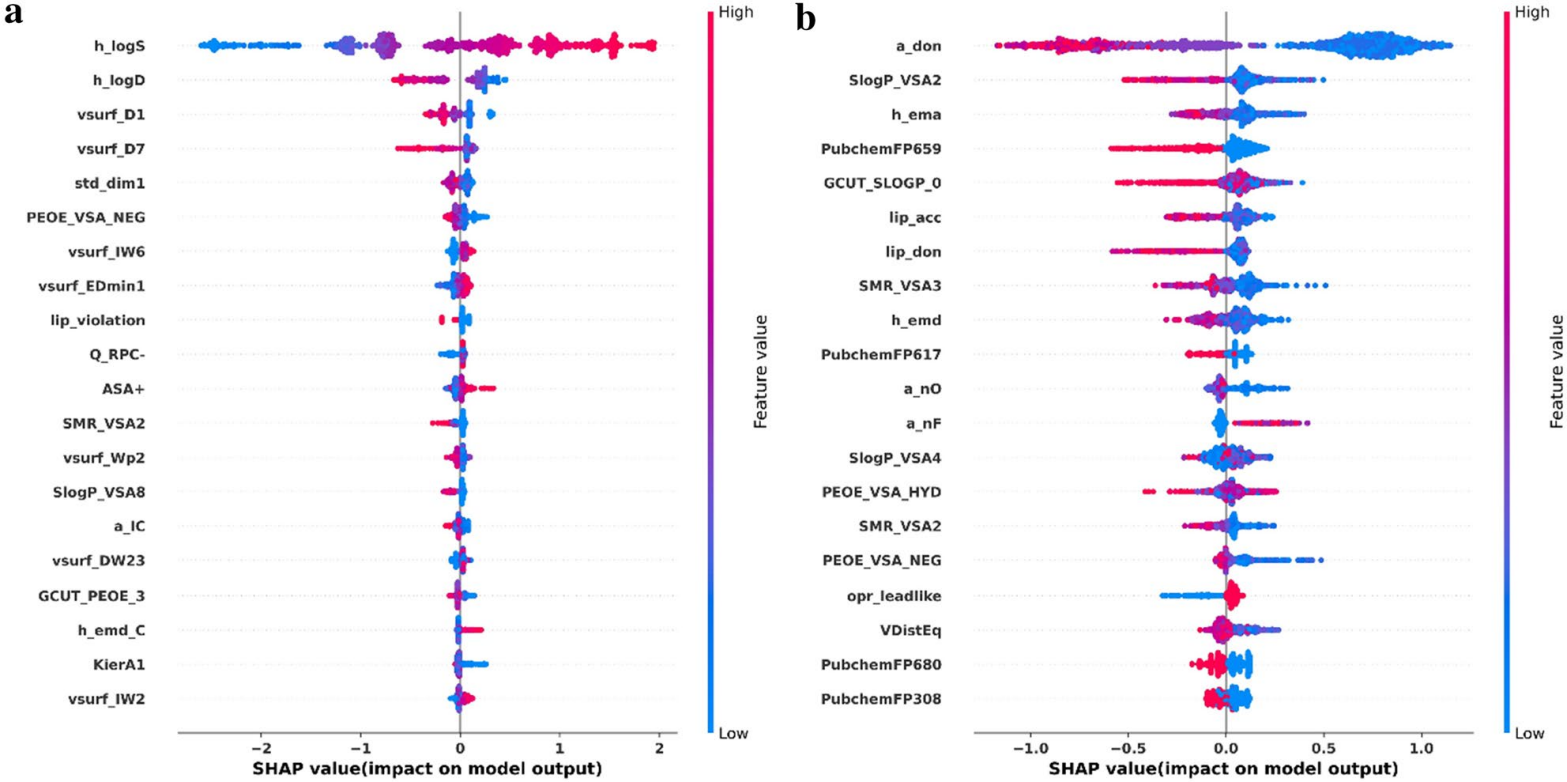
Importance of the representative molecular descriptors (the top 20) and the corresponding SHAP values given by XGBoost for the **a** ESOL and **b** BBBP datasets. One molecule gets one dot on each descriptor’s line and dots stack up to show density

To check whether the learned knowledge from XGBoost is interpretable and reasonable, the SHAP method was used to analyze and interpret the developed models. Here, the XGBoost models for a regression dataset (ESOL) and a classification dataset (BBBP) were used as the examples. The top 20 representative molecular descriptors and the corresponding SHAP values are presented in Fig. 2.

ESOL: ESOL is a small regression dataset for aqueous solubility. As can be seen from Fig. 2a, the most important descriptor given by the XGBoost model is h_logS, which represents the logarithm of aqueous solubility (mol/L). The feature value and SHAP value in Fig. 2a illustrate a clear positive correlation between the values of h_logS and the values of aqueous solubility, that means a higher h_logS will increase the aqueous solubility of a compound and vice versa, which is well in line with the expert knowledge. In Fig. 2a, h_logD (the octanol/water distribution coefficient at pH = 7), which is related to the hydrophobicity of molecules, is the second most important descriptor, and it presents a clear negative correlation with the value of aqueous solubility. This finding also well accords with the general phenomenon that higher hydrophobicity means lower solubility. In addition, the most significant parameter in the linear regression model for estimating the aqueous solubility of a compound developed by Delaney et al. is also a descriptor highly related to hydrophobicity (logP_octanol_) [71]. Other two significant descriptors, including vsurf_D1 and vsurf_D7 that measure the hydrophobic volume of a molecule, are highly related to hydrophobicity. Similar to h_logD, both of them have negative correlations with aqueous solubility, which is also well explainable where a higher hydrophobic volume will decrease the solubility of molecules.

#### BBBP

BBBP is a classification dataset for the blood–brain barrier (BBB) penetration of compounds. As we can see from Fig. 2b, a number of the representative descriptors show clearly inverse correlations with BBB permeability, especially the descriptors a_don, SlopP_VSA2, h_ema and PubchemFP659 (2-(methylamino)ethan-1-ol substurcture), implying higher values of such descriptors will block molecules to cross the BBB. Here, compared with the SHAP value distributions of other descriptors, that of opr_leadlike (Oprea’s lead-like test) shows a huge difference due to the clear and successive blue dots on the left part of Fig. 2b, indicating that opr_leadlike has positive correlations with BBB permeability. That’s to say, compounds with more lead-likeness would be more likely to cross the BBB. Here, most of those descriptors with inverse correlations with BBB permeability are polar-related descriptors, such as a_don (number of hydrogen bond donor atoms), h_ema (sum of hydrogen bond acceptor strengths) and PubchemFP659 (2-(methylamino)ethan-1-ol substurcture). This is consistent with the well-known fact that highly polar compounds have very low BBB permeation.

### Virtual screening profile analysis of different ML methods

**Fig. 3:**
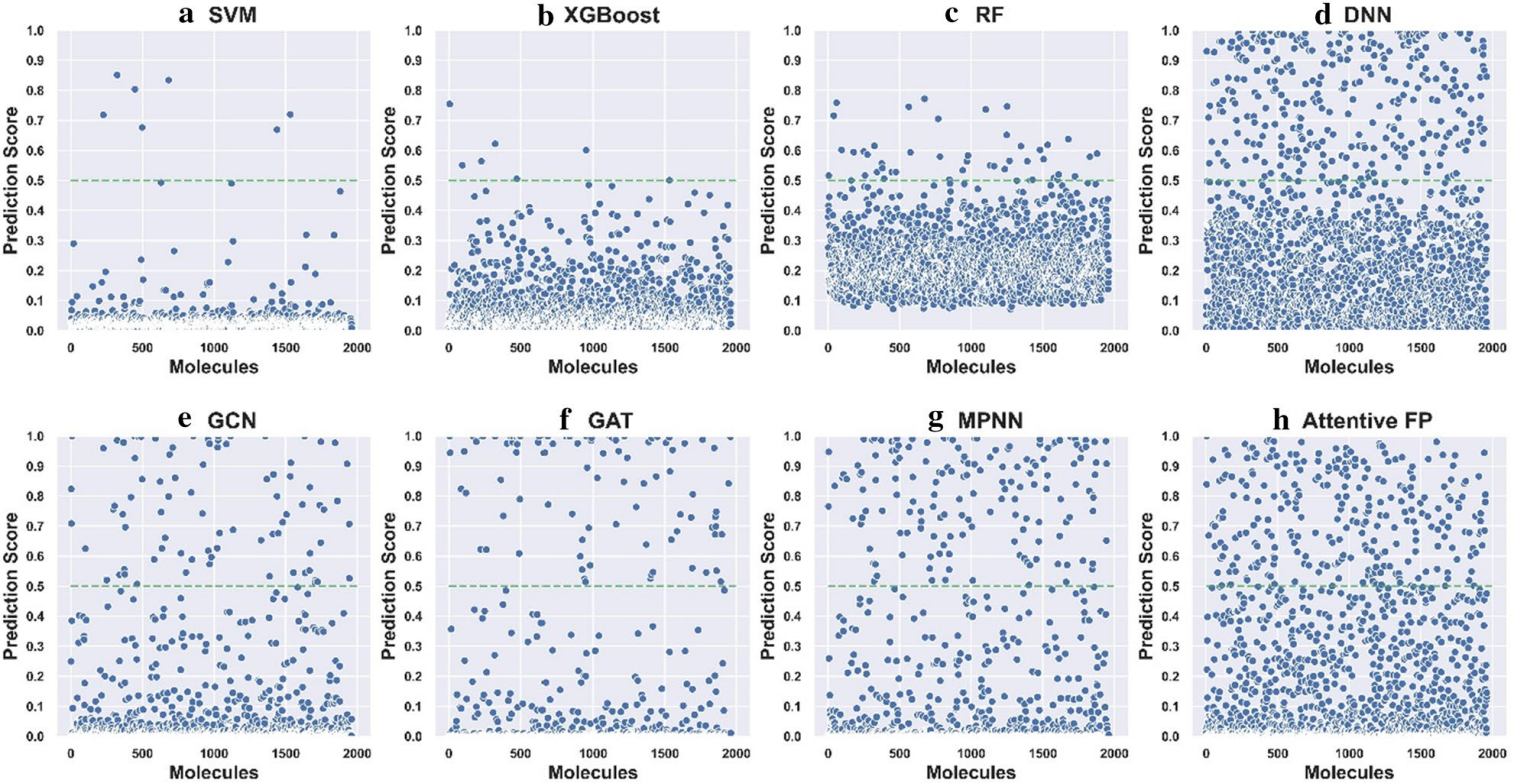
The distributions of the prediction scores for the 1960 screened molecules predicted by the four descriptor-based models including **a** SVM, **b** XGBoost, **c** RF, **d** DNN and the four graph-based models including **e** GCN, **f** GAT, **g** MPNN and **h** Attentive FP

Many efforts have been dedicated to improving the prediction accuracy of different ML algorithms for molecular property prediction. In reality, these models can be served as VS tools to search for potential candidates from large chemical libraries and promote the discovery process. In our opinion, the efforts to improve the predictive accuracy and explore the VS profiles of different ML methods have the same priority because different ML models may offer quite different predictions in practical VS campaigns even they have similar predictive accuracy, which may directly determine what kinds of candidates are experimentally tested. To this end, a case study was conducted by identifying potential inhibitors towards HIV replication through the four descriptor-based and four graph-based models, and the small molecule drugs deposited in DrugBank (Version: 5.1.5) were virtually screened by these models. All the explored models were developed based on the training set of the HIV dataset, optimized by the corresponding validation set and validated by the corresponding test set (the data folds were kept the same as those used in the first evaluation stage). The choice of this dataset was considered because of its relatively large data size and a more realistic proportion between inhibitors and noninhibitors. Prior to the screening, the polymers, inorganics, mixtures, salts were removed from the DrugBank small molecule drug database. The duplicated compounds between the DrugBank database and the training set were also eliminated from the database. Finally, the remaining 1960 small molecule drugs were used for screening. The output probability given by the optimal model was used as the score to measure the HIV replication inhibition ability (Fig. 3). The higher the prediction score is, the greater the likelihood of being a HIV inhibitor is, and vice versa.

**Fig. 4:**
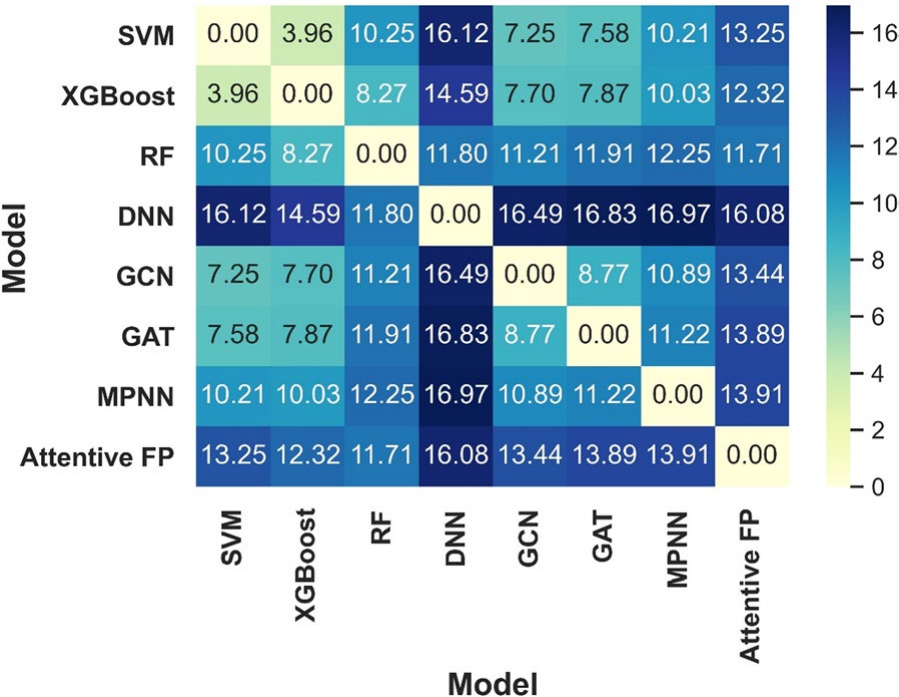
The heat map of the Euclidean distances of the prediction scores for different model pairs

It can be observed that the distributions of the prediction scores for the 1960 molecules given by the eight models vary from one to another although these models have similar prediction accuracy (Table 4). If an arbitrary threshold of 0.5 was used to classify inhibitors and non-inhibitors, the number of potential inhibitors given by the eight models are 7, 7, 45, 329, 86, 90, 158 and 284 respectively, highlighting the large difference of the predictions among different models. It seems that the conventional descriptor-based models (SVM, XGBoost and RF) are inclined to give more conservative predictions and the NN-based models are opposite. Among the eight models, SVM and XGBoost are the most two conservative models where only seven inhibitors were predicted by them. Inversely, the descriptor-based DNN model is the most radical one and about 17% compounds (329/1960) in the DrugBank database were predicted as inhibitors. Furthermore, the Euclidean distance of the prediction score distributions for the 1960 drugs given by any two models was used to investigate the VS profile similarity of model pairs, and the lower this distance is, the more similar between the VS profiles of two models is, and vice versa (the minimum and maximum of this distance here are 0 and 44.27, respectively). As shown in Fig. 4, with the exception of the SVM and XGBoost model pair, it is apparent that the Euclidean distances of the prediction scores between any two of the eight models are relatively high, demonstrating that different ML models could perform very differently in practical VS campaigns.

**Fig. 5:**
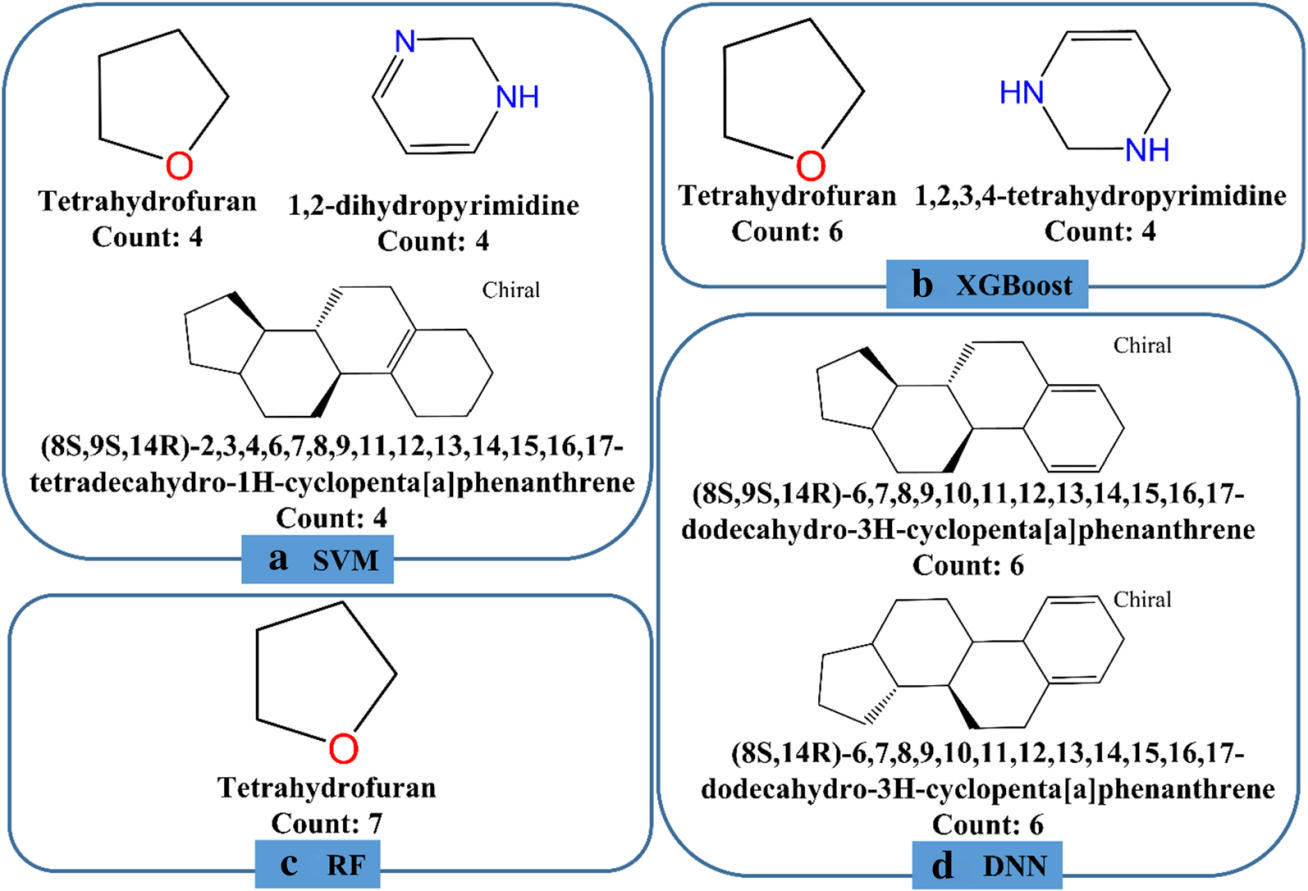
The structural features of the potential inhibitors given by the four descriptor-based models including **a** SVM, **b** XGBoost, **c** RF and **d** DNN

**Fig. 6:**
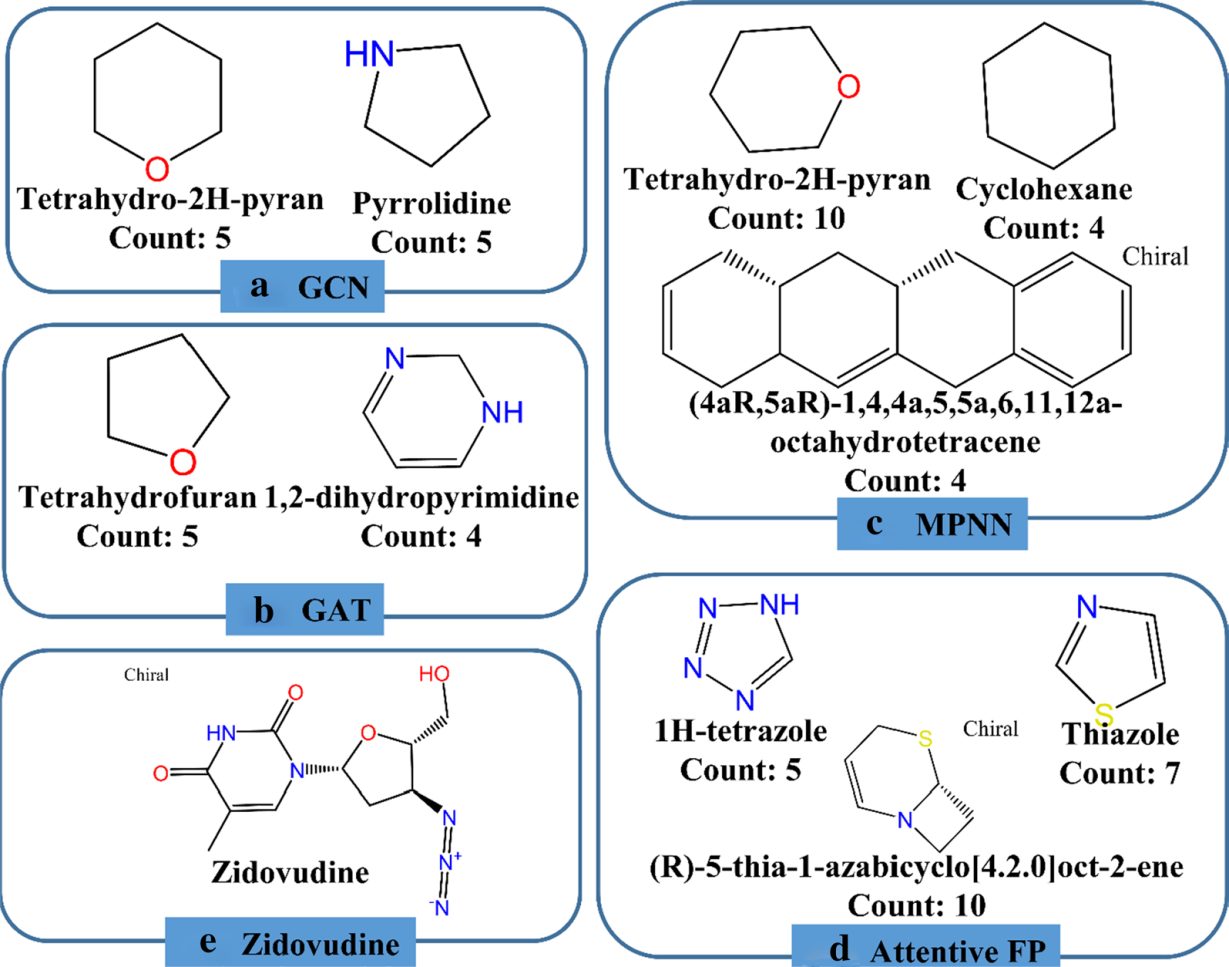
The structural features of the potential inhibitors predicted by the four graph-based models including **a** GCN, **b** GAT, **c** (MPNN) and **d** Attentive FP; **e** the structure of the known HIV inhibitor identified by all the eight models

In order to uncover the structural features of the potential HIV inhibitors predicted by different ML models. The top 20 compounds with the highest scores given by each model were decomposed into different structural fragments and analyzed using Pipeline Pilot 2017. Three types of structural fragments were used, including Murcko Assemblies (contiguous ring systems plus chains that link two or more rings), Ring Assemblies (contiguous ring systems), and Bridge Assemblies (contiguous ring systems that share two or more bonds). The generated fragments were counted and the representative fragments whose counts are higher than or equal to four (not consider the common benzene component) for each model are shown in Fig. 5 (descriptor-based models) and Fig. 6 (graph-based models). As expected, the structural features of the potential inhibitors given by different models are highly diverse, demonstrating that different ML models are inclined to identify different sets of candidates and their diverse performances may be contributed from the different features used in training and the different principles of the algorithms. In addition, in the top 160 compounds given by the eight ML models (20 compounds for each model), 116 compounds are unique, and only a small fraction of compounds (10) were ranked in the top 20 in any three models, which also supported the aforementioned argument. Among the 10 compounds, it is pleasurable to observe that one compound used to combat HIV/AIDS, zidovudine, was predicted as a promising HIV inhibitor by all the eight models (Fig. 6e). Here we found that the inhibitors predicted by the eight models share some nitrogen or oxygen heterocyclic components, four models including SVM, XGBoost, RF and GAT have the tetrahydrofuran component in their predicted inhibitors and two models including GCN and MPNN have the tetrahydro-2H-pyran component in their predicted inhibitors. The structural features given by the SVM and GAT models are highly overlapping. However, for all the eight ML models, no common structural component was found and the representative structural features given by the Attentive FP model show a high diversity. All in all, the structural features of the identified candidates by different ML models are diverse from each other.

### Washing results of the benchmark datasets

As described above, three washing steps were developed to automatically eliminate the incorrect or inappropriate structures from the original datasets. The washed datasets containing the original columns coupled with the canonical SMILES column were output as the final datasets. All of them are available in Additional file 1 and the detailed information of them are listed in Additional file 3: Table S5. As shown in Additional file 3: Table S5, several datasets, including BBBP, ClinTox, SIDER, Tox21, and ToxCast, contain relatively large numbers of incorrect or inappropriate structures (the ratio of the number of the removed compounds to its original number is large than 4%). In order to check the effect of the eliminated structures on model performance, two representative algorithms (i.e., XGBoost and Attentive FP) were used to build the prediction models for the washed datasets of BBBP, Tox21, ToxCast, and SIDER. The same hyper-parameters described above were used in model building. Similarly, the models were validated by 50 times independent runs and the statistical results are listed in Additional file 3: Table S6. It can be observed that the predictions of the models to the washed datasets do not show large difference compared with those to the original datasets. The predictions to the washed datasets of BBBP become slightly better for both models, while those to the washed datasets of ToxCast and SIDER become slightly worse for both models. And the predictions to the washed datasets of Tox21 get slightly better for XGBoost and slightly worse for Attentive FP. However, it should be noted that our purpose is not highlighting the impact of incorrect or inappropriate structures on the predictive accuracy of models but merely points out that the quality of the public datasets should be carefully checked.

## Conclusion

GNN has gained great interest in molecular property prediction due to its ability to learn molecular representations automatically. It appears that most studies reported so far have drawn the conclusion that GNN is more promising than traditional descriptor-based models. In this study, we demonstrated that on average the descriptor-based models outperform the graph-based models in the predictions of a variety of molecular properties in terms of predictive accuracy and computational efficiency. SVM generally gives the best predictions to regression tasks. Both XGBoost and RF can give reliable predictions to classification tasks, and graph-based methods, such as GCN and Attentive FP, can offer outstanding performance on a fraction of larger or multi-task datasets. In terms of computational efficiency, XGBoost and RF have fast computability and only need a few seconds to train a model even for a large dataset. Moreover, descriptor-based model can be well interpreted by the SHAP method. Finally, the ML models were used to conduct a VS study towards HIV, and the results demonstrate that different ML algorithms offer diverse VS profiles. In conclusion, our study illustrates that the descriptor-based models are able to achieve better or comparable predictions to the highly-intricate and specialized graph-based models in terms of prediction accuracy, computability and interpretability.

## Supplementary information

**Additional file 1.** The datasets for the single tasks.

**Additional file 2.** The python source codes that implement the workflow.

**Additional file 3: Table S1.** The performance of the top three runs and the worst three runs among the 50 times independent runs given by the XGBoost model for the 11 datasets. **Table S2**. The performance comparison (MAE metric) of the four descriptor-based and four graph-based models on the three regression datasets. **Table S3**. The performance comparison (R2 metric) of the four descriptor-based and four graph-based models on the three regression datasets. **Table S4:** The performance comparison (average RMSE) of the 50 times independent runs on three regression datasets including ESOL, FreeSolv, and Lipop before/after removing the top three related descriptors given by the four descriptor-based models (SVM, XGBoost, RF and DNN). All the models named with suffix ‘1’ refer to the models developed based on the remaining descriptors. **Table S5.** The detailed information for the 11 washed datasets. **Table S6.** The performance comparison of the 50 times independent runs on four datasets including BBBP, Tox21, ToxCast, and SIDER before/after washing for the XGBoost and Attentive FP models.
