# Supplementary material for: Could graph neural networks learn better molecular representation for drug discovery? A comparison study of descriptor-based and graph-based models
Source: J Cheminform. 2021 Feb 17;13:12. doi: 10.1186/s13321-020-00479-8 (PMC7888189; doi:10.1186/s13321-020-00479-8)
Supplement: Supplementary file 3 — Additional file 3: Table S1. The performance of the top three runs and the worst three runs among the 50 times independent runs given by the XGBoost model for the 11 datasets. Table S2. The performance comparison (MAE metric) of the four descriptor-based and four graph-based models on the three regression datasets. Table S3. The performance comparison (R2 metric) of the four descriptor-based and four graph-based models on the three regression datasets. Table S4: The performance comparison (average RMSE) of the 50 times independent runs on three regression datasets including ESOL, FreeSolv, and Lipop before/after removing the top three related descriptors given by the four descriptor-based models (SVM, XGBoost, RF and DNN). All the models named with suffix ‘1’ refer to the models developed based on the remaining descriptors. Table S5. The detailed information for the 11 washed datasets. Table S6. The performance comparison of the 50 times independent runs on four datasets including BBBP, Tox21, ToxCast, and SIDER before/after washing for the XGBoost and Attentive FP models. [file 13321_2020_479_MOESM3_ESM.docx]

**Supporting Materials**

**Table S1.** The performance of top three runs and worst three runs among the 50 times independent runs given by XGBoost for the 11 datasets.

| **Dataset** | **No.** | **Tasks** | **Metric** | **Model** | **Training** | **Validation** | **Test** |
| --- | --- | --- | --- | --- | --- | --- | --- |
| ESOL | 1127 | 1 | RMSE | XGBoost_Top3 | 0.195±0.062 | 0.568±0.077 | 0.489±0.009 |
|  |  |  |  | XGBoost_Worst3 | 0.218±0.020 | 0.549±0.011 | 0.708±0.036 |
| FreeSolv | 639 | 1 | RMSE | XGBoost_Top3 | 0.204±0.141 | 0.972±0.176 | 0.619±0.093 |
|  |  |  |  | XGBoost_Worst3 | 0.236±0.121 | 0.903±0.069 | 1.342±0.046 |
| Lipop | 4200 | 1 | RMSE | XGBoost_Top3 | 0.170±0.031 | 0.571±0.024 | 0.519±0.012 |
|  |  |  |  | XGBoost_Worst3 | 0.201±0.005 | 0.558±0.022 | 0.654±0.015 |
| BACE | 2035 | 1 | AUC_ROC | XGBoost_Top3 | 0.998±0.002 | 0.913±0.025 | 0.930±0.004 |
|  |  |  |  | XGBoost_Worst3 | 0.991±0.005 | 0.920±0.022 | 0.845±0.003 |
| BBBP | 1513 | 1 | AUC_ROC | XGBoost_Top3 | 0.997±0.002 | 0.936±0.014 | 0.975±0.005 |
|  |  |  |  | XGBoost_Worst3 | 0.991±0.004 | 0.951±0.010 | 0.875±0.009 |
| HIV | 40748 | 1 | AUC_ROC | XGBoost_Top3 | 0.998±0.002 | 0.841±0.011 | 0.858±0.011 |
|  |  |  |  | XGBoost_Worst3 | 0.982±0.011 | 0.822±0.028 | 0.780±0.004 |
| ClinTox | 1475 | 2 | AUC_ROC | XGBoost_Top3 | 0.975±0.003 | 0.922±0.046 | 0.966±0.006 |
|  |  |  |  | XGBoost_Worst3 | 0.991±0.005 | 0.953±0.010 | 0.841±0.010 |
| SIDER | 1366 | 27 | AUC_ROC | XGBoost_Top3 | 0.950±0.009 | 0.672±0.008 | 0.678±0.003 |
|  |  |  |  | XGBoost_Worst3 | 0.946±0.002 | 0.690±0.013 | 0.600±0.003 |
| Tox21 | 7811 | 12 | AUC_ROC | XGBoost_Top3 | 0.989±0.004 | 0.859±0.011 | 0.856±0.002 |
|  |  |  |  | XGBoost_Worst3 | 0.989±0.006 | 0.853±0.012 | 0.816±0.006 |
| ToxCast | 8539 | 182 | AUC_ROC | XGBoost_Top3 | 0.977±0.000 | 0.797±0.002 | 0.784±0.001 |
|  |  |  |  | XGBoost_Worst3 | 0.977±0.002 | 0.801±0.003 | 0.765±0.002 |
| MUV | 93087 | 17 | AUC_RRC | XGBoost _Top3 | 0.655±0.041 | 0.085±0.013 | 0.136±0.027 |
|  |  |  |  | XGBoost_Worst3 | 0.621±0.055 | 0.141±0.052 | 0.018±0.000 |

**Table S2.** The performance comparison (average MAE) of the 50 times independent runs on the three regression datasets for the eight models. (the top three model were bolded for each dataset).

| **Dataset** | **No.** | **Tasks** | **Metric** | **Model** | **Training** | **Validation** | **Test** |
| --- | --- | --- | --- | --- | --- | --- | --- |
| ESOL | 1127 | 1 | MAE | **SVM** | 0.108±0.002 | 0.404±0.033 | **0.408±0.037** |
|  |  |  |  | **XGBoost** | 0.170±0.044 | 0.422±0.033 | **0.425±0.036** |
|  |  |  |  | RF | 0.288±0.005 | 0.487±0.036 | 0.485±0.043 |
|  |  |  |  | DNN | 0.372±0.048 | 0.465±0.043 | 0.503±0.067 |
|  |  |  |  | GCN | 0.205±0.040 | 0.481±0.040 | 0.518±0.045 |
|  |  |  |  | GAT | 0.225±0.084 | 0.448±0.062 | 0.478±0.063 |
|  |  |  |  | MPNN | 0.350±0.058 | 0.488±0.034 | 0.518±0.053 |
|  |  |  |  | **Attentive FP** | 0.292±0.059 | 0.394±0.034 | **0.429±0.048** |
| FreeSolv | 639 | 1 | MAE | **SVM** | 0.139±0.005 | 0.477±0.099 | **0.493±0.096** |
|  |  |  |  | **XGBoost** | 0.169±0.115 | 0.611±0.097 | **0.637±0.096** |
|  |  |  |  | RF | 0.344±0.007 | 0.689±0.119 | 0.698±0.119 |
|  |  |  |  | **DNN** | 0.403±0.082 | 0.565±0.092 | **0.664±0.123** |
|  |  |  |  | GCN | 0.524±0.100 | 0.627±0.120 | 0.758±0.134 |
|  |  |  |  | GAT | 0.663±0.158 | 0.779±0.140 | 0.895±0.149 |
|  |  |  |  | MPNN | 0.580±0.151 | 0.767±0.148 | 0.878±0.152 |
|  |  |  |  | Attentive FP | 0.498±0.090 | 0.595±0.119 | 0.693±0.095 |
| Lipop | 4200 | 1 | MAE | **SVM** | 0.128±0.001 | 0.405±0.019 | **0.404±0.017** |
|  |  |  |  | **XGBoost** | 0.147±0.031 | 0.414±0.022 | **0.414±0.017** |
|  |  |  |  | RF | 0.373±0.003 | 0.504±0.022 | 0.501±0.019 |
|  |  |  |  | DNN | 0.209±0.052 | 0.429±0.018 | 0.444±0.019 |
|  |  |  |  | GCN | 0.267±0.050 | 0.456±0.024 | 0.475±0.022 |
|  |  |  |  | GAT | 0.284±0.064 | 0.493±0.026 | 0.505±0.028 |
|  |  |  |  | MPNN | 0.366±0.051 | 0.481±0.024 | 0.503±0.026 |
|  |  |  |  | **Attentive FP** | 0.233±0.035 | 0.383±0.019 | **0.392±0.018** |

**Table S3.** The performance comparison (average R2) of the 50 times independent runs on the three regression datasets for the eight models. (the top three model were bolded for each dataset).

| **Dataset** | **No.** | **Tasks** | **Metric** | **Model** | **Training** | **Validation** | **Test** |
| --- | --- | --- | --- | --- | --- | --- | --- |
| ESOL | 1127 | 1 | R2 | **SVM** | 0.995±0.000 | 0.925±0.014 | **0.924±0.015** |
|  |  |  |  | **XGBoost** | 0.988±0.007 | 0.923±0.016 | **0.921±0.017** |
|  |  |  |  | RF | 0.965±0.001 | 0.896±0.024 | 0.897±0.024 |
|  |  |  |  | DNN | 0.944±0.015 | 0.911±0.018 | 0.894±0.029 |
|  |  |  |  | GCN | 0.983±0.007 | 0.900±0.025 | 0.882±0.027 |
|  |  |  |  | GAT | 0.977±0.022 | 0.913±0.026 | 0.897±0.039 |
|  |  |  |  | MPNN | 0.950±0.017 | 0.900±0.020 | 0.886±0.023 |
|  |  |  |  | **Attentive FP** | 0.964±0.015 | 0.933±0.013 | **0.919±0.019** |
| FreeSolv | 639 | 1 | R2 | **SVM** | 0.994±0.001 | 0.952±0.023 | **0.948±0.021** |
|  |  |  |  | **XGBoost** | 0.995±0.009 | 0.929±0.030 | **0.924±0.029** |
|  |  |  |  | RF | 0.982±0.001 | 0.909±0.033 | 0.908±0.033 |
|  |  |  |  | **DNN** | 0.977±0.010 | 0.949±0.019 | **0.925±0.031** |
|  |  |  |  | GCN | 0.965±0.014 | 0.944±0.024 | 0.906±0.036 |
|  |  |  |  | GAT | 0.931±0.081 | 0.916±0.032 | 0.876±0.056 |
|  |  |  |  | MPNN | 0.951±0.028 | 0.909±0.031 | 0.874±0.046 |
|  |  |  |  | Attentive FP | 0.963±0.014 | 0.942±0.026 | 0.915±0.030 |
| Lipop | 4200 | 1 | R2 | **SVM** | 0.975±0.001 | 0.775±0.028 | **0.770±0.035** |
|  |  |  |  | **XGBoost** | 0.974±0.011 | 0.773±0.025 | **0.773±0.032** |
|  |  |  |  | RF | 0.842±0.003 | 0.695±0.026 | 0.701±0.031 |
|  |  |  |  | DNN | 0.946±0.028 | 0.762±0.025 | 0.745±0.034 |
|  |  |  |  | GCN | 0.906±0.043 | 0.734±0.034 | 0.692±0.085 |
|  |  |  |  | GAT | 0.900±0.045 | 0.695±0.036 | 0.678±0.062 |
|  |  |  |  | MPNN | 0.841±0.042 | 0.713±0.034 | 0.688±0.035 |
|  |  |  |  | **Attentive FP** | 0.932±0.022 | 0.800±0.025 | **0.789±0.029** |

**Table S4**: The performance comparison (average RMSE) of the 50 times independent runs on three regression datasets including ESOL, FreeSolv, and Lipop before/after removing the top three related descriptors given by the four descriptor-based models (SVM, XGBoost, RF and DNN). All the models named with suffix ‘1’ refer to the models developed based on the remaining descriptors.

| **Dataset** | **No.** | **Tasks** | **Metric** | **Model** | **Training** | **Validation** | **Test** |
| --- | --- | --- | --- | --- | --- | --- | --- |
| ESOL | 1127 | 1 | RMSE | SVM | 0.149±0.005 | 0.565±0.038 | 0.569±0.052 |
|  |  |  |  | SVM1 | 0.224±0.006 | 0.579±0.046 | **0.586±0.057 (↓)** |
|  |  |  |  | XGBoost | 0.224±0.057 | 0.573±0.048 | 0.582±0.056 |
|  |  |  |  | XGBoost1 | 0.159±0.048 | 0.574±0.054 | **0.582±0.067 (↓)** |
|  |  |  |  | RF | 0.391±0.008 | 0.664±0.053 | 0.663±0.074 |
|  |  |  |  | RF1 | 0.410±0.008 | 0.673±0.062 | **0.682±0.079 (↓)** |
|  |  |  |  | DNN | 0.492±0.061 | 0.617±0.060 | 0.670±0.092 |
|  |  |  |  | DNN1 | 0.484±0.063 | 0.616±0.068 | **0.665±0.087 (↑)** |
| FreeSolv | 639 | 1 | RMSE | SVM | 0.307±0.023 | 0.804±0.192 | 0.852±0.171 |
|  |  |  |  | SVM1 | 0.323±0.024 | 0.807±0.193 | **0.865±0.173 (↓)** |
|  |  |  |  | XGBoost | 0.228±0.168 | 0.988±0.197 | 1.025±0.185 |
|  |  |  |  | XGBoost1 | 0.314±0.132 | 0.944±0.212 | **0.953±0.177 (↑)** |
|  |  |  |  | RF | 0.518±0.011 | 1.129±0.248 | 1.143±0.230 |
|  |  |  |  | RF1 | 0.582±0.020 | 1.181±0.269 | **1.184±0.231 (↓)** |
|  |  |  |  | DNN | 0.574±0.115 | 0.840±0.158 | 1.013±0.197 |
|  |  |  |  | DNN1 | 0.581±0.144 | 0.844±0.166 | **1.045±0.218 (↓)** |
| Lipop | 4200 | 1 | RMSE | SVM | 0.191±0.005 | 0.566±0.037 | 0.577±0.039 |
|  |  |  |  | SVM1 | 0.266±0.005 | 0.582±0.038 | **0.591±0.040 (↓)** |
|  |  |  |  | XGBoost | 0.191±0.040 | 0.569±0.033 | 0.574±0.034 |
|  |  |  |  | XGBoost1 | 0.169±0.030 | 0.576±0.030 | **0.582±0.033 (↓)** |
|  |  |  |  | RF | 0.478±0.003 | 0.660±0.031 | 0.659±0.031 |
|  |  |  |  | RF1 | 0.403±0.004 | 0.652±0.031 | **0.654±0.030 (↑)** |
|  |  |  |  | DNN | 0.271±0.068 | 0.583±0.031 | 0.608±0.034 |
|  |  |  |  | DNN1 | 0.276±0.060 | 0.583±0.031 | **0.608±0.031 (↑)** |

**Table S5.** The detailed information for the 11 washed data sets.

| **Dataset** | **Original No.^a^** | **Uncovered^b^** | **Inorganics** | **Duplicates^c^** | **New No.** |
| --- | --- | --- | --- | --- | --- |
| **BACE** | 1513 | 0 | 0 | 1 | 1510 |
| **BBBP** | 2050 | 0 | 1 | 11 | 1963 |
| **ClinTox** | 1484 | 7 | 23 | 42 | 1360 |
| **ESOL** | 1128 | 0 | 0 | 6 | 1110 |
| **FreeSolv** | 642 | 0 | 3 | 0 | 639 |
| **HIV** | 41127 | 6 | 33 | 16 | 40905 |
| **Lipophilicity** | 4200 | 0 | 0 | 9 | 4182 |
| **MUV** | 93087 | 0 | 0 | 2 | 93083 |
| **SIDER** | 1427 | 0 | 47 | 25 | 1318 |
| **Tox21** | 7831 | 8 | 74 | 112 | 7445 |
| **ToxCast** | 8597 | 20 | 99 | 291 | 7728 |

^a^The number is counted from MoleculeNet, and all the washing operations are based on the original datasets reported by Wu *et al*.^1^ ^b^Compounds cannot be recognized by MOE or RDKit; ^c^Duplictaes with inconsistent labels.

**Table S6.** The performance comparison of the 50 times independent runs on four datasets including BBBP, Tox21, ToxCast, and SIDER before/after washing for the XGBoost and Attentive FP models.

| **Dataset** | **Model** | **Tasks** | **Training** | **Validation** | **Test** |
| --- | --- | --- | --- | --- | --- |
| **BBBP** | XGBoost (No. 2035) | 1 | 0.995±0.005 | 0.938±0.022 | 0.926±0.026 |
|  | XGBoost (No. 1963) |  | 0.997±0.005 | 0.937±0.017 | **0.931±0.020 (↑)** |
|  | Attentive FP (No. 2035) |  | 0.972±0.021 | 0.922±0.027 | 0.887±0.032 |
|  | Attentive FP (No. 1963) |  | 0.977±0.030 | 0.925±0.020 | **0.901±0.028 (↑)** |
| **Tox21** | XGBoost (No. 7811) | 12 | 0.989±0.005 | 0.857±0.009 | 0.836±0.010 |
|  | XGBoost (No. 7445) |  | 0.991±0.005 | 0.858±0.011 | **0.843±0.009 (↑)** |
|  | Attentive FP (No. 7811) |  | 0.939±0.021 | 0.859±0.012 | 0.852±0.012 |
|  | Attentive FP (No. 7445) |  | 0.929±0.018 | 0.857±0.014 | **0.850±0.012 (↓)** |
| **ToxCast** | XGBoost (No. 8539) | 182 | 0.976±0.002 | 0.800±0.004 | 0.774±0.004 |
|  | XGBoost (No. 7728) |  | 0.977±0.003 | 0.799±0.012 | **0.772±0.012 (↓)** |
|  | Attentive FP (No. 8539) |  | 0.921±0.037 | 0.804±0.020 | 0.794±0.017 |
|  | Attentive FP (No. 7728) |  | 0.890±0.045 | 0.790±0.020 | **0.776±0.020 (↓)** |
| **SIDER** | XGBoost (No. 1366) | 27 | 0.954±0.010 | 0.694±0.023 | 0.642±0.020 |
|  | XGBoost (No. 1318) |  | 0.945±0.009 | 0.682±0.021 | **0.636±0.017 (↓)** |
|  | Attentive FP (No. 1366) |  | 0.834±0.103 | 0.657±0.024 | 0.623±0.026 |
|  | Attentive FP (No. 1318) |  | 0.849±0.110 | 0.646±0.028 | **0.615±0.025 (↓)** |

**References**

(1) Wu, Z.; Ramsundar, B.; Feinberg, E. N.; Gomes, J.; Geniesse, C.; Pappu, A. S.; Leswing, K.; Pande, V. MoleculeNet: a benchmark for molecular machine learning. *Chemical Science* **2018,** *9*, (2), 513-530.
